# QbD-Based Design Space Development for Honey-Containing Traditional Chinese Medicine Tablets Assisted by the SeDeM Expert System and Machine Learning

**DOI:** 10.3390/pharmaceutics18081014

**Published:** 2026-08-16

**Authors:** Xinxin Deng, Dandan Mu, Fei Song, Yeqing Miao, Qiang Yin, Hailong Yin

**Affiliations:** 1 Research Institute of Xinjiang Uyghur Pharmaceutical Co., Ltd., Pharmacy College of Shihezi University, Shihezi 832003, China; 20242115016@stu.shzu.edu.cn; 2 Xinjiang Uygur Pharmaceutical Co., Ltd., Urumqi 830026, China; mdandan2025@163.com (D.M.); fay55555555@163.com (F.S.); 18399027834@163.com (Y.M.)

**Keywords:** SeDeM, machine learning, quality by design, design space, honey, TCM tablets

## Abstract

**Background/Objectives**: Oral solid dosage forms of traditional Chinese and ethnic medicines are currently undergoing modernisation. The objective of this study is to explore the scope for formulation variation arising from batch-to-batch fluctuations in intermediates, and to identify the factors influencing key quality attributes of honey-containing tablets. In this regard, a machine-learning-based predictive model is being formulated that will integrate and analyse formulation factors and the results characterised by the SeDeM expert system. Utilising the SeDeM index as a mediating variable, the study endeavours to establish a comprehensible and predictable stepwise research pathway to provide a foundation for industrial-scale upscaling. **Methods**: Twelve SeDeM expert systems were utilised to characterise honey-containing granules for formulation screening, to evaluate their suitability for use in traditional Chinese medicine honey-containing tablet systems, and to identify key limiting factors and the feasibility space affecting the quality of the final product; Based on the QBD philosophy, a TriAD (Tri-criterion Adaptive Design) design scheme was proposed, integrating the horizontal balance of orthogonal designs, the spatial coverage of uniform designs, and the parameter estimation efficiency of D-optimal designs into the experimental layout of the formulation feasibility space; Through further data aggregation, a multi-layer feature set comprising four formulation factors, six SeDeM indicators, and three critical quality attributes (CQAs) was constructed. The mediating effects of the SeDeM indicators were revealed through different pathways involving 37 combinations of simple, linear, and Bootstrap models. Furthermore, 180 linear and non-linear machine learning models (comprising 12 categories of algorithms) were trained to predict formulation and CQA outcomes, ultimately completing the design space mapping and validation. **Results**: The results show that the SeDeM parameters effectively bridge the CQA results of different honey formulations, with these indicators acting as selective mediators between formulation factors and CQAs. Compared with a pure data model relying solely on raw formulation variables, the introduction of SeDeM knowledge, combined with high-information-content samples obtained via TriAD, improved the predictive performance and robustness of the SeDeM–ML hybrid model in terms of disintegration time and hardness; its R^2^_LOO increased by 0.267 and 0.510, respectively, and the overall predictive space was significantly expanded. Experimental validation was conducted using formulations within the design space predicted by the optimal model; the results showed that both the prediction bias and the relative standard deviation were less than 5 percent. **Conclusions**: The present study demonstrates that SeDeM can not only be used to evaluate formulations of honey-containing TCM tablets but also serves as an intermediary bridge linking formulation factors, granule-mechanism variables, and tablet quality outcomes. TriAD, in turn, further translates the QbD philosophy into an actionable formulation space design, thereby providing a development pathway for honey-containing tablets that combines interpretability, predictability, and QbD consistency, and offers new insights for the industrial application of oral TCM preparations.

## 1. Introduction

Transforming traditional compound formulations into contemporary preparations suitable for industrial production is the fundamental step in modernizing oral preparations of traditional Chinese medicine and ethnic medicines. The design of the manufacturing process and the choice of dosage forms have always been crucial to the development. Yangxin Dawa Yimixike honey paste is a traditional medicine that includes 26 medicinal ingredients such as musk, sandalwood, cassia bark, and pearl, and is traditionally used for the treatment of chest pain, palpitations, gastric weakness, and neurasthenia. It was selected as the model formulation in this study because its honey content and complex multicomponent matrix make tablet conversion technically challenging, while honey, as an indispensable excipient, cannot be readily removed or replaced. There is no scientific explanation for the mechanisms underlying the stability of honey-based traditional Chinese medicine treatments, even though traditional experience forms the basis for their development. Controlling how changes in compound intermediates affect the final product’s quality is even more challenging. In addition, the modernization of traditional Chinese medicine should involve not only manufacturability and quality consistency, but also evidence-based safety evaluation. Recent TEC-based studies further highlight the importance of integrating chemical, biological, and mechanistic evidence into this process [1]. This restriction is especially noticeable when creating honey-based formulations. Zuo & Xu et al. investigated honey-containing systems in early-stage production processes and in contemporary Chinese herbal medicine formulations [2,3]. The assessment of additional requirements for honey tablets in Chinese herbal medicine was summarized in Zheng and Han’s review. The results of this research and regulatory practice mostly indicate that conventional dose forms, such as honey tablets and water-honey pills, are the main focus of current literature and regulatory practice [4]. However, systematic research on the use of honey extraction as a crucial variable in tablet design is limited. The difficulty with honey-containing tablets stems not only from the honey’s variable viscosity but also from the fact that honey is not a “single excipient.” According to Dai, Choi, and Verpoorte, honey can be used in Traditional Chinese Medicine as an excipient in formulation preparation, an auxiliary material in herb processing, and a stand-alone medicinal component [5]. Chen, Ye, and Zhu further emphasized in their review of research on the preparation of medicinal materials using liquid excipients that the role of liquid excipients goes far beyond simply making the manufacturing process easier. They can change the transformation of medicinal constituents, dissolution behavior, pharmacokinetic processes, and even the manifestation of therapeutic effects [6]. Liu et al.’s work, which used Morus alba bark as a model, shows that processing herbal materials with honey results in systematic alterations in their overall chemical composition [7]. As a result, honey cannot be simply classified as a “binder” at the formulation level; rather, it should be considered a complicated formulation variable that simultaneously affects the powder’s final quality, flow characteristics, compressibility, lubricity, and hygroscopicity. Therefore, a more basic question must be addressed to manufacture honey-containing tablets: as a crucial intermediary variable, how does honey affect the end product’s quality when mixed with other excipients? Is it possible to quantify these mechanisms and use the results to guide tablet formulation design for honey-containing tablets?

The ability of the SeDeM expert system to quantify complex particle behavior using a set of interpretable signs is what makes it valuable. Suñé-Negre, Aguilar-Díaz, Scholtz, and others have shown that SeDeM can effectively support formulation design and has good predictive capability for direct compression formulations across different combinations of excipients and active pharmaceutical ingredients (APIs) in chemical pharmaceuticals [8,9,10]. This technique is not restricted to traditional small-molecule medications, as evidenced by Wan et al.’s application to a combination of Rhodiola extract and excipients [11]. Meanwhile, SeDeM is currently mostly focused on direct compression systems for chemical drugs and is rarely used in traditional Chinese medicine; the experiments presented in this paper are necessary to confirm its applicability and predictive accuracy in complex powdered systems containing honey. More significantly, the development of Chinese medicine tablets containing honey must examine the nonlinear mechanism by which changes in intermediates are transferred to the final product’s quality, rather than methods that aim for local optimality in stabilizing the material properties of chemical pharmaceutical preparations. Although the QbD framework provides a theoretical paradigm for this [12,13], the traditional response surface method, due to its implicit assumption of low-order or nearly linear relationships, is unable to effectively capture the complex nonlinear mappings among formulation variables, powder behavior, and quality attributes in the honey-containing system. Therefore, it is necessary to develop a new modeling strategy that can adapt to the complexity of the honey-containing systems in traditional Chinese medicine, based on the verification of the applicability of SeDeM. Recent studies in other engineering fields have shown that AI-driven frameworks can efficiently explore high-dimensional design spaces and support multivariable optimization [14]. Meanwhile, machine-learning research has emphasized that feature selection in multi-output systems should consider both factor-response relationships and interactions among response variables [15]. These advances are highly relevant to honey-containing traditional Chinese medicine tablets, where formulation factors, powder properties, and tablet CQAs are strongly interdependent.

This paper proposes a SeDeM-TriAD-ML fusion framework specifically designed for honey-containing traditional Chinese medicine pills to address the previously described application issues. This methodology reconfigures the design space as a pharmaceutically feasible area, simultaneously constrained by SeDeM particle characterization and intermediate qualities, rather than treating it as a simple statistical sampling zone. First, we will quantitatively characterize particle compressibility using the 12 SeDeM properties. Furthermore, we may determine how the SeDeM system and prescription factors interact to affect CQA by modeling the intermediate layer. Following that, determine which spatial domain best combines controllability and interpretability. In this framework, the synergistic management of several excipients can, on the one hand, compensate for the inherent fluctuations in traditional Chinese medicine intermediates, thereby ensuring the consistency of the final product. On the other hand, a foundation for control can be established with an eye toward industrial-scale production by defining acceptable ranges for key attributes of the intermediates. In terms of experimental design, the recently suggested TriAD design strategy improves sample quality during the modeling phase and region exploration efficiency during the experimental phase by facilitating the effective acquisition of high-information-content samples within the feasible region identified by the SeDeM gating mechanism. The development of tablets has shifted in recent years from empirical screening to predictive formulation, driven by machine learning research: Abbas et al. integrated a digital recipe system [16], Ghazwani and Hani used AI to predict disintegration times [17], and Hamaguchi et al. performed multi-index prediction [18]. In order to guide the modeling process towards the mechanisms underlying pharmaceutical formulation, this study integrates and analyzes experimental data from the SeDeM expert system and TriAD, testing their mediating roles in formulation development through different pathways using 37 combinations of simple linear and Bootstrap models. By training and evaluating 180 permutations of linear and non-linear machine learning models across 12 algorithm categories, we were able to further predict and validate the design space. This study’s main goal is to determine whether it is feasible to integrate traditional powder characterization, contemporary experimental design, and machine learning into a formulation optimization framework that balances interpretability and predictive accuracy for large-formulation traditional Chinese medicine tablets in which honey is used in the formulation process. This will give a transferable methodological paradigm for enhancing dose forms and promoting smart manufacturing in traditional Chinese and ethnic medicines, as well as a data-driven foundation for the product’s industrial-scale upscaling.

## 2. Materials and Methods

### 2.1. Materials

Yangxin Dawa Yimixike intermediates are manufactured by Xinjiang Uyghur Pharmaceutical Co., Ltd., Urumqi, China. Microcrystalline cellulose, used as a filler, was purchased from Anhui Shanhe Pharmaceutical Excipients Co., Ltd., Huainan, China; starch was purchased from Changling Jilong Bio-Pharmaceutical Co., Ltd., Songyuan, China; lactose was purchased from Zhenjiang Kangfu Bio-Engineering Co., Ltd., Zhenjiang, China; and dextrin was purchased from Changling Jilong Bio-Pharmaceutical Co., Ltd. The refined honey, used as a binder, was produced by Xinjiang Uyghur Pharmaceutical Co., Ltd. Sodium carboxymethyl starch, used as a disintegrant, was sourced from Huzhou Zhanzhan Pharmaceutical Co., Ltd., Huzhou, China; cross-linked polyvinylpyrrolidone from Anhui Shanhe Pharmaceutical Excipients Co., Ltd.; starch from Changling Jilong Biopharmaceutical Co., Ltd.; magnesium stearate, used as a lubricant, from Huzhou Zhanzhan Pharmaceutical Co., Ltd.; and magnesium stearate, used as a flow aid, from Anhui Shanhe Pharmaceutical Excipients Co., Ltd. All samples in this study were prepared by the authors themselves. The granulation process utilised the wet granulation method to ensure batch consistency. All raw materials were weighed on an analytical balance and uniformly mixed in accordance with the formulation requirements. The methods used to add the extract and refined honey were consistent, and the granulation process was standardised. The electric heating air-drying oven (model HN101-2, Nantong Huinan Scientific Instrument Co., Ltd., Nantong, China) was used for drying. The values of each particle parameter after drying were measured using the corresponding instruments. After measurement, the samples were mixed according to the corresponding formula (with the mixing time held constant). After mixing, the samples were compressed by the rotary tablet press (model VFP-7, Changzhou Longcheng Chenguang Pharmaceutical and Chemical Machinery Co., Ltd., Changzhou, China) with fixed specifications and pressure. After each sample group was prepared, the associated instruments were used to measure each core tablet parameter.

### 2.2. SeDeM Measurement Method

According to the SeDeM framework, powder properties are categorized into five groups of influencing elements based on their functional features and are generally characterized by 12 essential parameters [19]. Bulk density (Da) and tapped density (Dc) are dimensional parameters primarily used to describe the packing behavior and volume change properties of powders. Interparticle porosity (Ie), the Carr index (IC), and the cohesion index (Icd) are examples of compressibility parameters that represent the compressive behavior of powders, the interparticle pore structure, and their capacity to form tablets. During the production process, the flow and packing qualities of powders are assessed using flowability metrics such as the Hausner ratio (IH), angle of repose (α), and flow time (t″). Loss on drying (%HR) and hygroscopicity (%H) [20], which are mainly connected to the material’s and the tablets’ moisture sensitivity and storage stability, are factors that affect lubricity and stability. Particle-size distribution, the proportion of fine particles, and dosage [19] uniformity are characterized by dose-related parameters such as the uniformity index (Iθ) and the percentage of particles smaller than 75 μm (%Pf). The key coefficients for converting the experimental values (V) to radius values (r) were identified through a thorough analysis of the aforementioned parameters, based on the specific measurement results for each parameter, as well as the calculation formulas and conversion techniques outlined in Table 1. For detailed methodological information, see the Supplementary Materials section. When there are 12 test parameters, fusually taken as 0.952. It is generally accepted that, provided that IP≥0.5, IPP≥5 and IGC≥5, the powder can be deemed suitable for direct tableting [21]. In a study on pediatric carbamazepine orodispersible tablets, the researchers lowered the SeDeM incidence radius from 5.0 to 4.0–3.5 and were still able to produce tablets that met the predetermined critical quality attributes, demonstrating that the value of 5.0 commonly used in the SeDeM expert system is not an absolute threshold that cannot be changed [22]. Depending on the prescription and the unique qualities of the variety, this threshold can be suitably lowered. Nonetheless, it continues to be the best assessment method available for investigating and modifying the tablet production process. The precise measuring techniques for each parameter are listed below. The results were then displayed graphically based on the corresponding experimental findings.

### 2.3. CQA Determination

#### 2.3.1. Determination of the Disintegration Time

The test was conducted in accordance with the provisions of the Chinese Pharmacopoeia, Volume IV (2025 Edition), 0921: Disintegration Time Test (Rising and Falling Disintegration Apparatus).

#### 2.3.2. Determination of Hardness

To characterize the mechanical strength of the tablets, a hardness tester was used to test each formulation with 10 units. The testing method followed the general procedure specified in Chapter 2.9.8 of the European Pharmacopoeia.

#### 2.3.3. Determination of Friability

The test was conducted using a friability tester in accordance with the provisions of the Chinese Pharmacopoeia, Volume IV (2025 Edition), under the “Friability Test for Tablets” section.

### 2.4. Progressive Experimental Design

#### 2.4.1. Preliminary Screening of Excipients

##### Screening of Major Excipients

Honey-containing traditional Chinese medicine tablets contain a certain proportion of viscous components, which often leads to insufficient flowability, unstable compaction behavior, and sticking during direct compression. Therefore, empirical selection of fillers and disintegrants is associated with considerable uncertainty. In the present study, the SeDeM expert system was employed to screen and compare excipients, including fillers and disintegrants. At this stage, the intermediate and refined honey were combined with different candidate fillers (major excipients) to prepare granules, and the complete set of SeDeM parameters was determined for each formulation. Based on the calculated comprehensive indices, including the parameter index (IP), parameter profile index (IPP), and good compressibility index (IGC), the effects of different major excipient combinations on powder flowability, compressibility, and suitability for tableting were comparatively evaluated. The specific screening parameters are listed in Table S1 (For detailed information, see the Supplementary Materials section).

Following preparation according to the above formulation design, the SeDeM parameters of each formulation were determined. Subsequently, the disintegrant, glidant, and lubricant were incorporated in fixed proportions, and the mixtures were compressed into tablets. The CQAs of the resulting tablets were then evaluated. At this stage, meeting the predefined acceptance criteria for all CQAs was considered the minimum requirement for selecting the formulation. The IGC was used as the primary comparative index, and the radial values of individual parameters were also considered to select the excipient combination with the best overall performance as the basis for subsequent formulation studies.

##### Screening of Disintegrants

Based on the major excipient combination selected in Section Screening of Major Excipients, the proportions of the preliminarily screened major excipients were retained, and the disintegrants listed in Table S2 (For detailed information, see the Supplementary Materials section) were incorporated into the formulations, while the levels of the glidant and lubricant were kept constant. The optimal disintegrant was selected based on lower friability, shorter disintegration time, and higher hardness as the primary evaluation criteria.

#### 2.4.2. Factor-Level FFD Experimental Design

The dose ranges for the important formulation parameters were examined further once the principal filler and disintegrant types were identified. If optimization tests are conducted directly inside a large factor space during formulation development, there is a significant experimental effort and a high probability of producing several combinations that are not practical for tableting. Consequently, this study utilised the SeDeM evaluation before the formal optimisation process to preliminarily delineate the feasible range of prescription variables. At this stage, the excipient types identified during the preliminary screening were finalised, and the focus was on examining four formulation factors: the proportion of refined honey (X1), the dosage of the disintegrant (X2), the moisture content (X3), and the dosage of MCC (X4). Among these, the amount of refined honey may influence the powder’s binding properties, moisture content, and ability to form tablets after compression [2]. The dosage of disintegrant is closely related to the disintegration behaviour and pore structure of the tablets [23]. Residual moisture may alter the wettability of powders and interparticle forces [24]. MCC, a commonly used direct compression excipient, is highly compressible and helps with disintegration; it is frequently used to improve the tableting properties of powders [25].

This study employed a partial factorial design with a resolution of 2^(4-1) to investigate the effects of four formulation factors on the suitability of honey-containing powder for direct tableting. The design used X4 = X1 × X2 × X3 as the generative element, with a total of eight factorial points and three centre points. The eight factor points are primarily used to estimate the main effects of each factor, whilst the three centre points are used to detect any curvature effects that may be present and to estimate experimental error. The experimental sequence was randomised to minimise the impact of operational sequence, environmental variations, and fluctuations in material condition on the experimental results [26] (see Table S3 for detailed information; see the Supplementary Materials section).

#### 2.4.3. TriAD Experimental Design

The process parameters were further defined and optimized based on the range obtained following convergence and the primary factor evaluation in Section 2.4.2. At this point, the emphasis shifted from comparing formulation points for individual factors to analyzing the quantitative relationships between sample critical quality attributes (CQAs) and key process parameters (CPPs) to define a multidimensional design space with operational flexibility. This study presents the Tri-criterion Adaptive Design (TriAD) for the first time, based on experimental needs and leveraging the benefits of conventional design-space exploration schemes (see Table S4 for detailed information; see the Supplementary Materials section). To guarantee balanced factor-level allocation, improve design space coverage, and boost the effectiveness of model parameter estimation, this design incorporates three conventional experimental schemes [27,28,29]. While the D-optimal criterion can improve the quality of the information matrix and reduce the variance of parameter estimates, TriAD can minimize the interference of factor correlations in estimating main effects and improve the model’s applicability across the entire design space by filling in missing data points [30]. This helps to establish robust CPPs-CQAs. Regarding the analysis of mediating effects, TriAD provides greater experimental variation in the path relationships among CPPs, mediating variables, and CQAs, thereby facilitating the distinction between CPPs’ direct effects on CQAs and their indirect effects mediated by powder properties or SeDeM indices. Consequently, this design not only establishes the design space but also provides a data foundation for subsequent elucidation of mechanisms and pathway analysis.

The specific experimental design is shown in Table S5 (For detailed information, see the Supplementary Materials section), in which Group A comprises eight axial points positioned along the axes of each factor, with coordinates of (±1, 0, 0, 0), (0, ±1, 0, 0), (0, 0, ±1, 0) and (0, 0, 0, ±1). These points extend the orthogonal design structure, enabling the estimation of purely quadratic effects, which is crucial for characterising the curvature of response surfaces and identifying the optimal operating region [31]. Group B consists of 12 supplementary internal points, designed to enhance the uniformity and coverage of the sample distribution within the four-dimensional design space; the axial distance α is set to 1.0 rather than a rotational value, ensuring that all experimental points remain within the validated feasible region. This prioritisation of practical feasibility over theoretical rotatability is consistent with QbD principles; it enhances spatial uniformity—as measured by deviation metrics—by ensuring that experimental points are distributed within the interior of the design space rather than solely at the vertices and along the axes [32]. The six additional points in Part C are selected according to the D-optimal criterion, i.e., by choosing the combination of experimental points from the set of candidates that maximises |X′X| to reduce the generalised variance of the parameter estimates [33]. A candidate set can be generated within the design space using Latin hypercube sampling; the selected set of points is then iteratively updated with a permutation algorithm to exclude points already included in the design [34]. This method ensures that the points in Part C fill the gaps left by the structured components (A and B), whilst improving both spatial coverage (the uniformity objective) and information content (the D-optimal objective).

### 2.5. Comparison of Data Analysis and ML Modelling

The original data matrix was constructed by integrating the factor-level FFD experimental design described in Section 2.4.2 with the TriAD experimental design described in Section 2.4.3. Each formulation run comprised 13 variables: X(4), SeDeM(5 + 1), and Y(3). Specifically, the four formulation factors (X) were treated as independent variables; the five SeDeM dimensions, together with the IGC, were treated as intermediate variables; and the CQAs (Y) were treated as dependent variables. Different modeling approaches were then applied to the data to evaluate, from multiple perspectives, the interpretability and predictive performance of the SeDeM system for formulating honey-containing traditional Chinese medicine tablets and their CQAs.

This organization was chosen because the SeDeM variables were intended to capture the changes in powder behavior through which formulation adjustments propagate to tablet quality; they were therefore treated as mechanistically intermediate descriptors rather than as additional end-point responses. The initial regression models were restricted to main effects to provide an interpretable first-order description and to avoid over-parameterization for a dataset of this size. Quadratic terms and two-factor interactions were introduced only when they were statistically supported and formulation-relevant. Accordingly, the simplified models were used primarily to interpret the direction and relative magnitude of factor effects and to assess the bridging role of SeDeM variables. In contrast, predictive conclusions were confined to the formulation domain covered by the combined FFD and TriAD experiments.

#### 2.5.1. Evaluation of Linear Models for Factor Levels, the Five SeDeM Dimensions, and IGC and CQA (Simplified Model Analysis)

A simplified regression model was developed using pooled data from 37 data points to describe the relationship between process parameters (CPPS) and the SeDeM indices and the key quality attributes of the tablets. For details on model construction, see Table 2. The model was constructed based on a main effects model, with quadratic and interaction terms that were statistically significant and of pharmaceutical significance being progressively added [35]. Model selection takes into account a combination of adjusted R2, LOOCV R2, RMSECV, VIF, residual diagnostics, and model interpretability [36,37]; see Table S6 (For detailed information, see the Supplementary Materials section). Given the limited sample size, the model’s predictive performance was evaluated using leave-one-out cross-validation (LOOCV). In each iteration, one experimental point was omitted, the model was developed using the remaining 36 points, and the omitted point was then predicted. After all 37 iterations were completed, the cross-validation metrics were calculated using the aggregated predictions [38]. Optimal simplification equations were established separately for the IGC, the five SeDeM sub-indices, and Y1, Y2 and Y3. For CQAs, further models were developed: a direct model from CPPs to CQAs; a path model from CPPs to SeDeM indicators; a path model from SeDeM indicators to CQAs; and a joint model in which CPPs and SeDeM indicators jointly predict CQAs. This modelling approach can be used not only to identify key influencing factors but also to provide a basis for assessing the potential mediating role of the SeDeM indicator between CPPs and CQAs.

#### 2.5.2. Analysis of Mediating Effects

The modeling in this phase will focus on determining whether the SeDeM dimensions influence the relationship between prescriptions and quality, as SeDeM is a crucial evaluation framework in this study. Determine which SeDeM dimension is the most significant mediating factor. Examine whether the effects of interactions between prescription factors are mediated as well. Provide a theoretical foundation for implementing Quality by Design. The path analysis paradigm put forward by Baron and Kenny [39] is used to investigate mediating effects, and the Bootstrap approach is used to generate confidence intervals for indirect effects [40,41]. As indirect effects do not usually satisfy the assumption of normality, bootstrap confidence intervals are considered more robust than traditional tests based on a normal approximation [40,41]; see Table 3 for specific settings. The primary goal of this study’s mediation analysis is to investigate the SeDeM index’s potential mediating role; mechanistic insights, rather than rigid causal conclusions, are the main focus of the interpretation of the results. Assumptions about the timing of variables, model specification, and correction for unmeasured confounders also affect the validity of the causal mediation study [42].

#### 2.5.3. Machine Learning Modelling Methods

To further evaluate the combined predictive ability of formulation factors, manufacturing process factors, and SeDeM sub-indices for the key quality attributes of tablets, this study introduced machine-learning regression models. Machine learning modelling focuses on improving predictive accuracy and identifying non-linear relationships, interactions between variables, and local similarities. Despite having different goals, these two methods function well together. For example, data on intricate pharmaceutical formulations and manufacturing processes have been analyzed using comparative mechanism analysis and a predictive modeling framework [35]. As shown in Table 4, this study created two primary types of feature techniques to compare conventional data-driven modeling with modeling that incorporates pharmaceutical formulation knowledge. In this case, SeDeM was used as an input, in addition to the original formulation factors and polynomial characteristics, and as a stand-alone predictor.

This study selected linear, regularized, local learning, kernel, and tree-ensemble models for comparison to prevent bias in the findings arising from using a single model structure. Table 5 displays the specifics of the model library. To ensure comparability of the outcomes, all models were evaluated using the same data split and evaluation measures.

A separate predictive model is established for each CQA, covering Y1 (disintegration time), Y2 (hardness), and Y3 (fragility). To minimise the impact of random partitioning on the results, K-fold cross-validation was employed. For models sensitive to feature scaling, including Ridge, Lasso, Elastic Net, Bayesian Ridge, KNN, and SVR, scaling parameters were estimated using only the training data, and the same transformation was subsequently applied to the corresponding validation or test data to prevent data leakage. Polynomial feature expansion was likewise performed exclusively within the training workflow, and cross-validation was conducted using a unified modeling pipeline. Model performance was evaluated using R^2^, RMSE, and MAE, where R^2^ quantifies the proportion of variance explained by the model, RMSE is more sensitive to large errors, and MAE reflects the average absolute prediction error [53].

#### 2.5.4. Design Space Prediction

Following the comparative evaluation of machine learning models, this stage involves constructing a design space based on the optimal prediction model, with a view to addressing the issues inherent in traditional prescription optimisation—namely, the focus on identifying a single optimal point and the difficulty in accounting for the operational range and risks associated with process fluctuations. According to the definition of ‘Design Space’ in ICH Q8(R2), the design space is not a single optimal formulation point, but rather a multidimensional operating region within which, for a given range of input variables, all key quality attributes can be simultaneously maintained within their predefined specifications. Consequently, at this stage, X is used as the input variable, and a four-dimensional grid search is performed within its operational range; the machine learning model optimised in Section 2.5.3 is then utilised to predict the disintegration time (Y1), hardness (Y2), and friability (Y3). Each candidate formulation must satisfy both the CQA requirements and the SeDeM sub-index thresholds to be included in the design space. The four-dimensional design space is primarily visualised using a two-dimensional projection; key CQA or SeDeM indicators that constrain the design space are identified through constraint bottleneck analysis, and, within the design space, the objective function and robustness analysis are employed to screen and select recommended formulations. See Table 6 for particular methodological specifics. This approach offers a more reliable foundation for subsequent prescription selection than single-point optimization, as it can address questions such as which prescription combinations are feasible, which constraints define the design space, whether the suggested prescription lies far from the failure boundary, and which variables require the most control.

#### 2.5.5. Validation Experiments

Based on the design space validation results, three formulations were selected for further validation. The deviations between the validation batches and the predicted CQA values were compared to ascertain the reliability of the model’s predictions and to verify the validity of the design space. This ultimately confirmed the feasibility of the SeDeM-ML integrated framework as a method for supporting design space research.

## 3. Results and Discussion

### 3.1. Results of Excipient Screening

#### 3.1.1. Results of Major Excipients Screening

**Figure 1:**
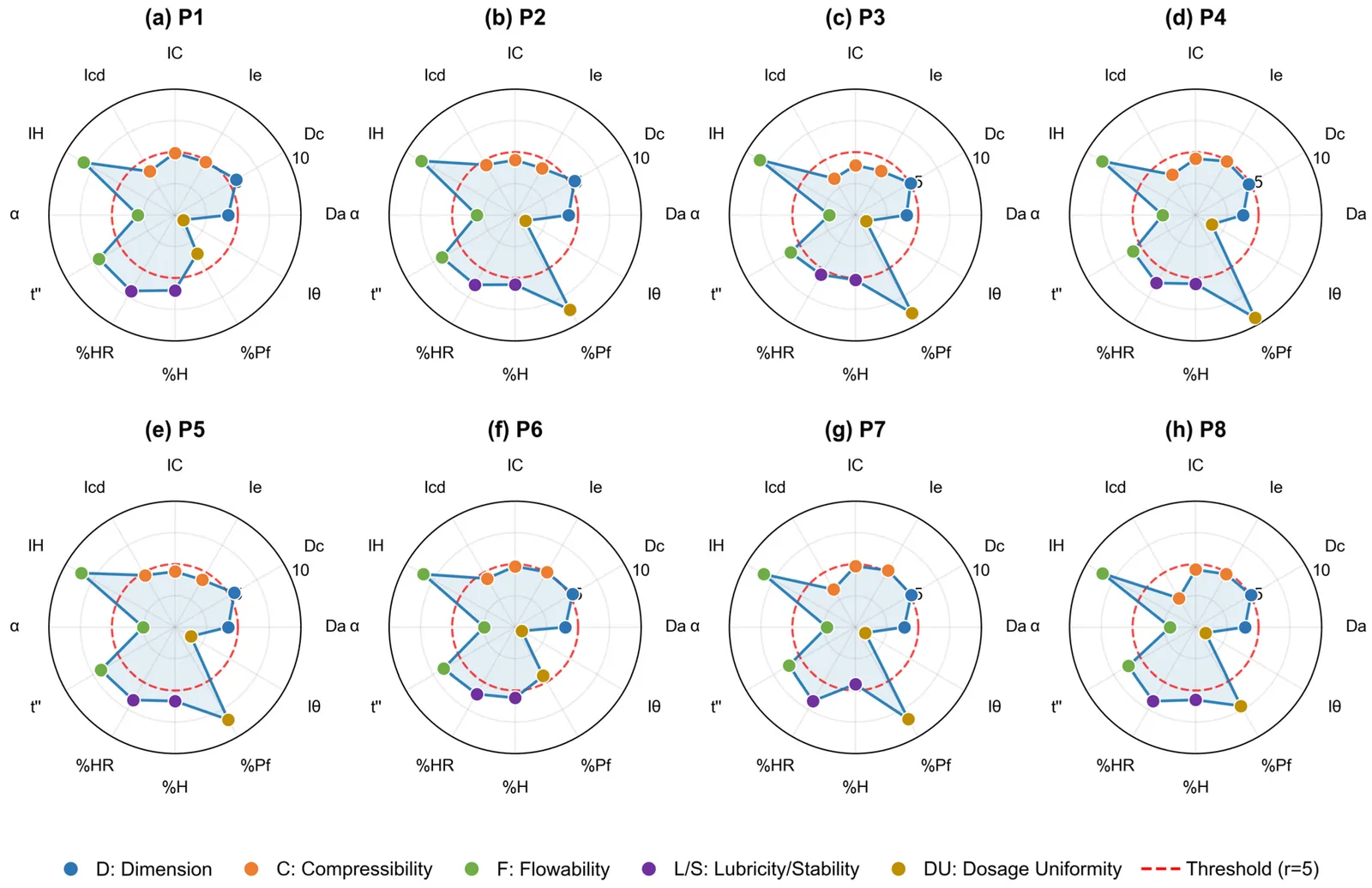
Comparative SeDeM radar plots for excipient screening; The blue polygon represents the overall SeDeM profile of each formulation. The red dashed circle indicates the acceptance threshold (r = 5). Parameters located outside the threshold are considered acceptable, whereas those inside the threshold indicate attributes that may require further optimization.

Granules were produced using several filler combinations in accordance with the aforesaid process, and the pertinent parameters were identified. The 12 SeDeM parameters were further integrated into radar plots (Figure 1) for comprehensive evaluation. Subsequently, tablets were produced by external excipient filling, and the corresponding critical quality attributes (CQAs) were obtained. The results are summarized in Table 7.

During the initial screening of excipients, IGC is used as the primary comparative indicator to assess the extent to which different excipient combinations improve the direct-compression properties of honey-containing powder tablets. As shown in the table, the IGC for formulation P5—which uses a combination of composite dextrin and microcrystalline cellulose as excipients—exceeds 5; however, using dextrin and microcrystalline cellulose alone still yields relatively favourable results. It can also be observed that, for traditional Chinese medicine granules containing honey, regardless of the excipients selected, the IGC for direct compression is unlikely to exceed the acceptable threshold of 5.0 during the granulation stage alone. Therefore, before tableting, the mixture must be blended with additional excipients to improve tableting performance. The results of a comprehensive evaluation, based on mixing additives in the same proportions and CQA analysis, are shown in Table 8. It should be noted here that IGC does not replace the final quality assessment of tablets; rather, it serves as a screening tool in pre-formulation studies, designed to minimise blind trials and improve the targeted selection of excipients.

The main excipients with superior overall performance—dextrin and composite microcrystalline cellulose—were selected based on a thorough CQA assessment, and their ratios were subsequently adjusted.

#### 3.1.2. Results of the Disintegrant Screening

Following confirmation of the main auxiliary ingredients, various disintegrants were evaluated; the results are shown in Table 9:

The disintegrants were evaluated based on three comprehensive criteria: disintegration time < 20 min, hardness > 50 N, and friability < 0.1 percent. P9 yielded excellent results, P10 was acceptable, and P11 performed poorly; consequently, PVPP from P9 was ultimately selected as the disintegrant for the formulation.

### 3.2. Evaluation of the Feasible Region for FFD Factor Ranges

**Figure 2:**
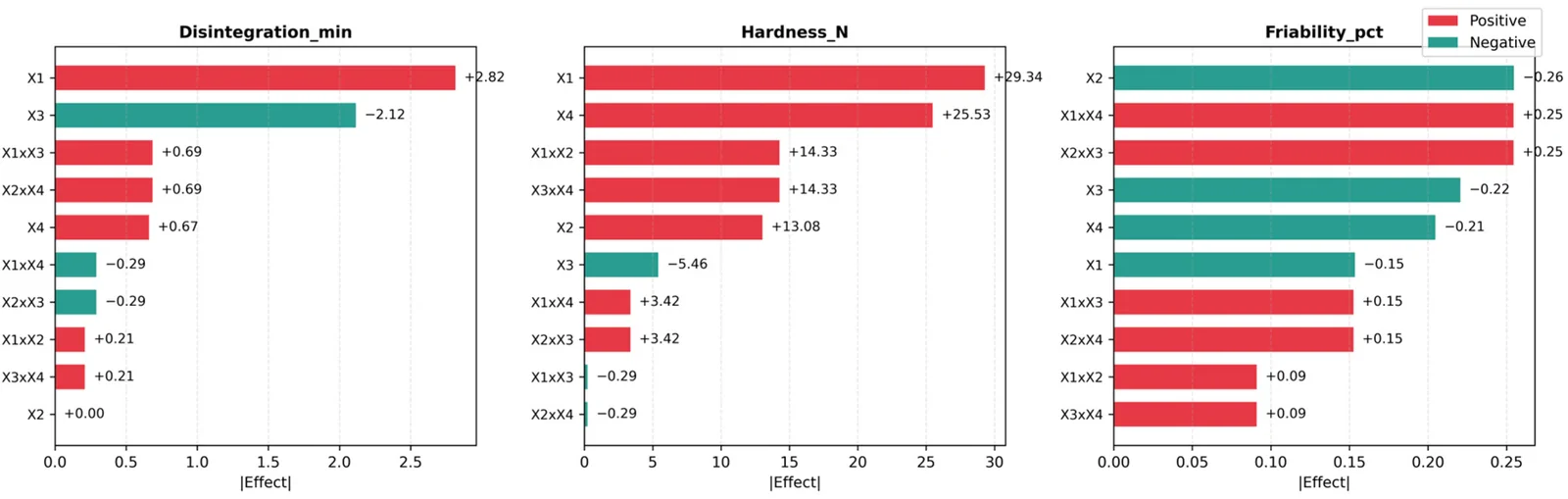
Pareto charts showing the magnitude and direction of the main and interaction effects of formulation variables on disintegration time, hardness, and friability.

**Figure 3:**
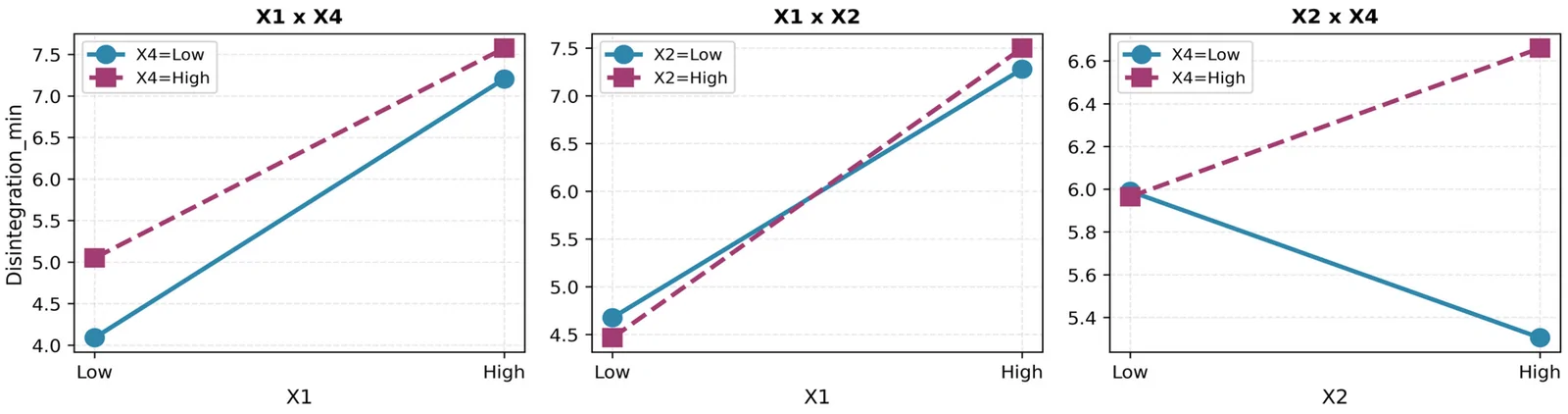
Interaction plots showing the combined effects of formulation variables on disintegration time at low and high factor levels.

**Figure 4:**
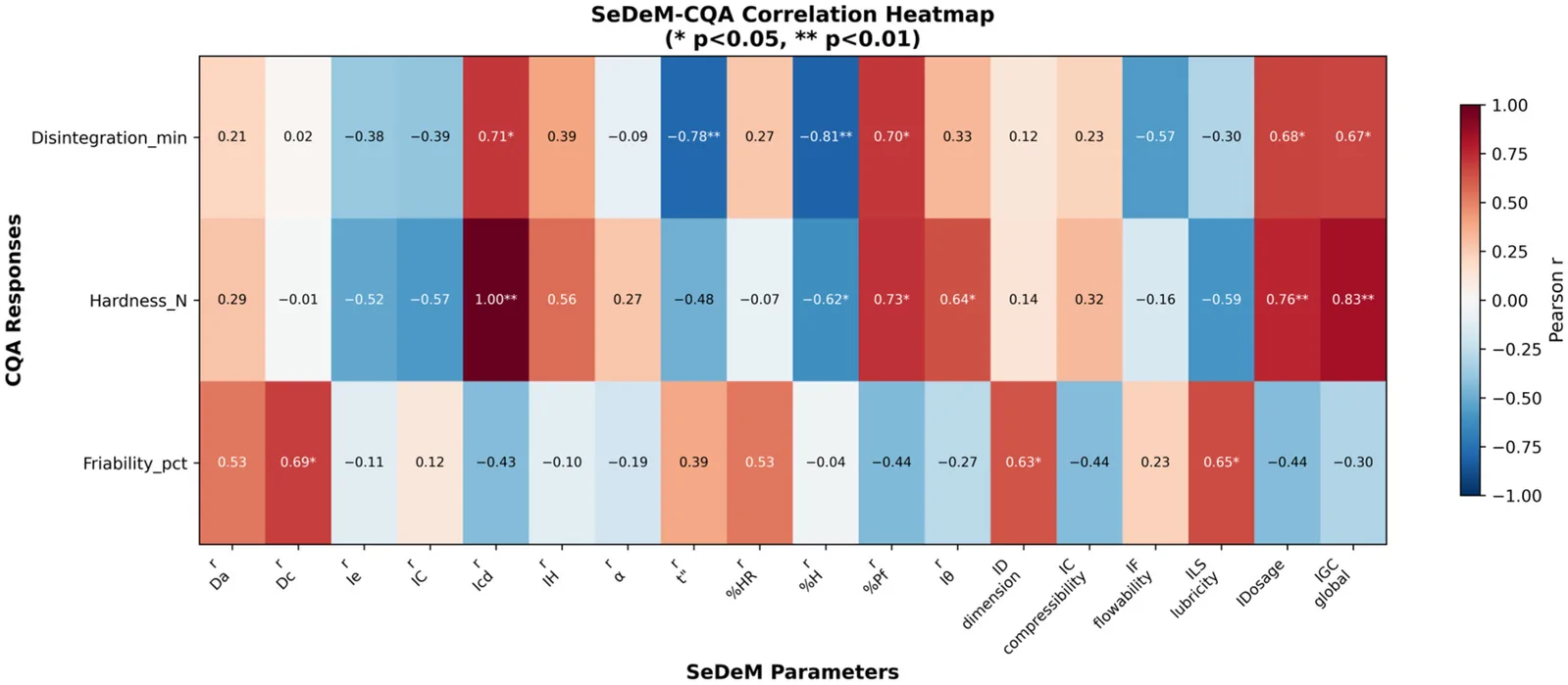
Correlation heatmap showing the Pearson correlation coefficients between SeDeM parameters and CQAs, with color indicating correlation direction and magnitude and asterisks indicating statistical significance.

Based on the selected types of auxiliary materials, this stage examined the effects of the following factors: X1: 20–30%; X2: 2–6%; X3: 2–6%; X4: 40–80%, on the five-dimensional parameters of SeDeM, IGC, and CQA results (see Table 10). Furthermore, Figure 2, Figure 3 and Figure 4 provide a comprehensive analysis of the relationships between these three factors and the key influencing factors.

According to experimental results and FFD factor analysis, X1 had a significant effect on IGC. Hardness tests showed that higher honey concentrations produce a degree of plastic deformation different from that observed in conventional tablets, despite raising IGC values. The amount of honey in the tablet formulation must be adjusted because this may affect long-term stability. At the same time, Figure 2 shows that, with respect to disintegration time, X1 (honey) is the most significant factor, followed by X4 (MCC) and the X1 × X4 interaction; X3 (moisture content) and X2 (disintegrant) have the least effect. Figure 3 shows that X1 × X4 exhibits a strong interaction, indicating that during process optimisation, no single factor can be optimised in isolation. In Figure 4, ID, ILS, IDosage and IGC show a high positive correlation of varying degrees with all three CQAs, confirming that the SeDeM system can effectively predict the critical quality attributes of honey-containing tablets. Collectively, these findings highlight the broader challenge of controlling powder properties and tablet performance in formulations containing complex natural materials. Different strategies may be adopted to address this challenge. For example, pulsed fluidized-bed agglomeration has been reported to improve the handling properties of pre-gelatinized riceberry powder through particle-structure modification [58]. By contrast, the present study employed SeDeM-guided, QbD-based formulation optimization to improve the flowability, compressibility, and manufacturability of honey-containing TCM powders through rational excipient selection and predictive modeling. Thus, particle engineering and formulation design represent complementary approaches to improving the processability and product quality of natural material-based tablet systems.

The workable zone for this stage has thus been reduced to X1: 20–28%; X2: 2–6%; X3: 2–6%; and X4: 40–70% based on the aforementioned analysis. After that, we’ll explore space to find a better formulation method.

### 3.3. TriAD Experimental Results

SeDeM parameters and CQA measurements were carried out using the TriAD experimental design factors; the results are shown in Table 11. Each group comprises three levels of information: X, SeDeM and Y. Further comprehensive modelling and analysis were conducted by aggregating these data with similar data from the FFD.

### 3.4. Evaluation of the Interrelationships Between Factor Levels, the Five Dimensions of SeDeM, and IGC and CQA

**Figure 5:**
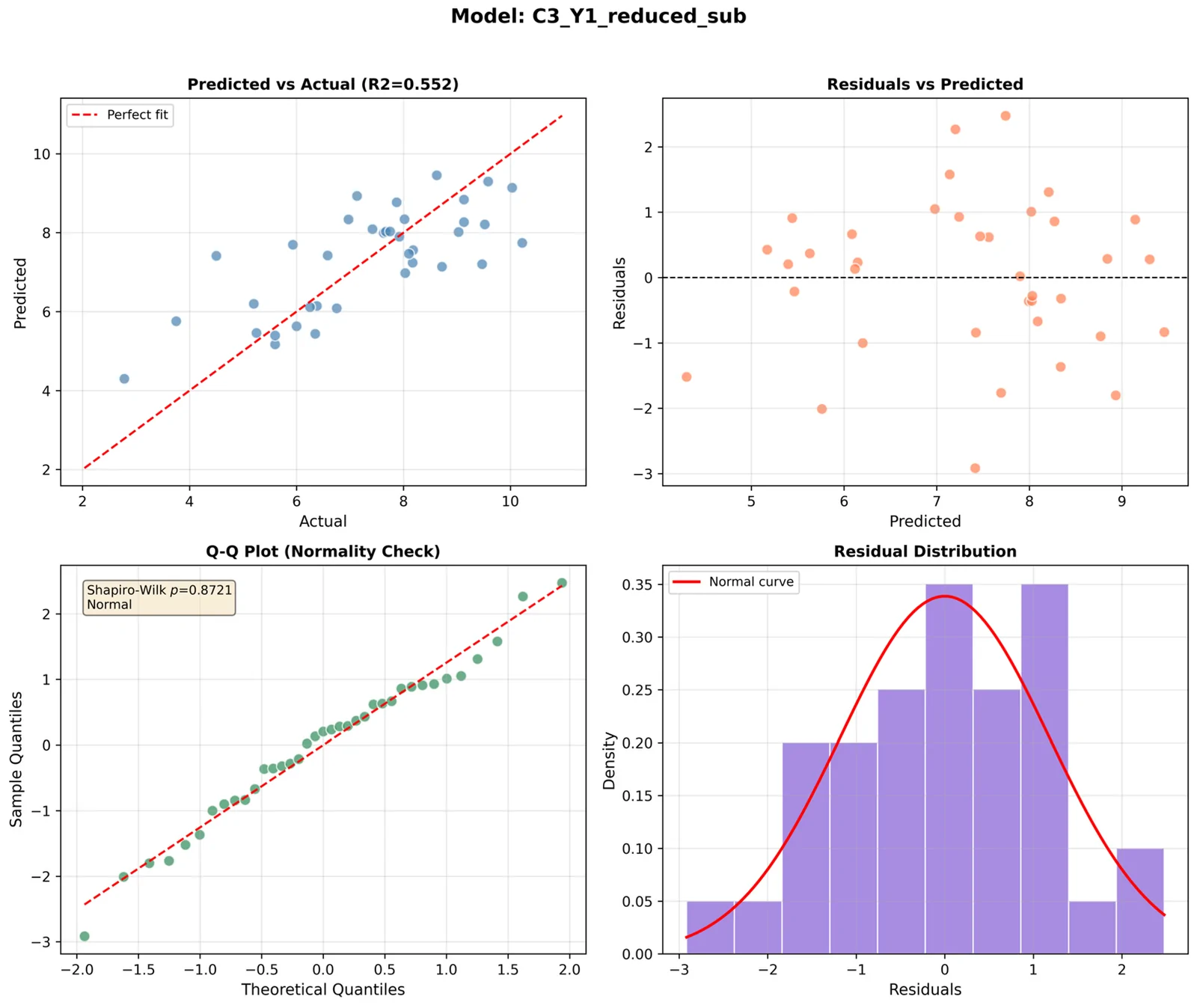

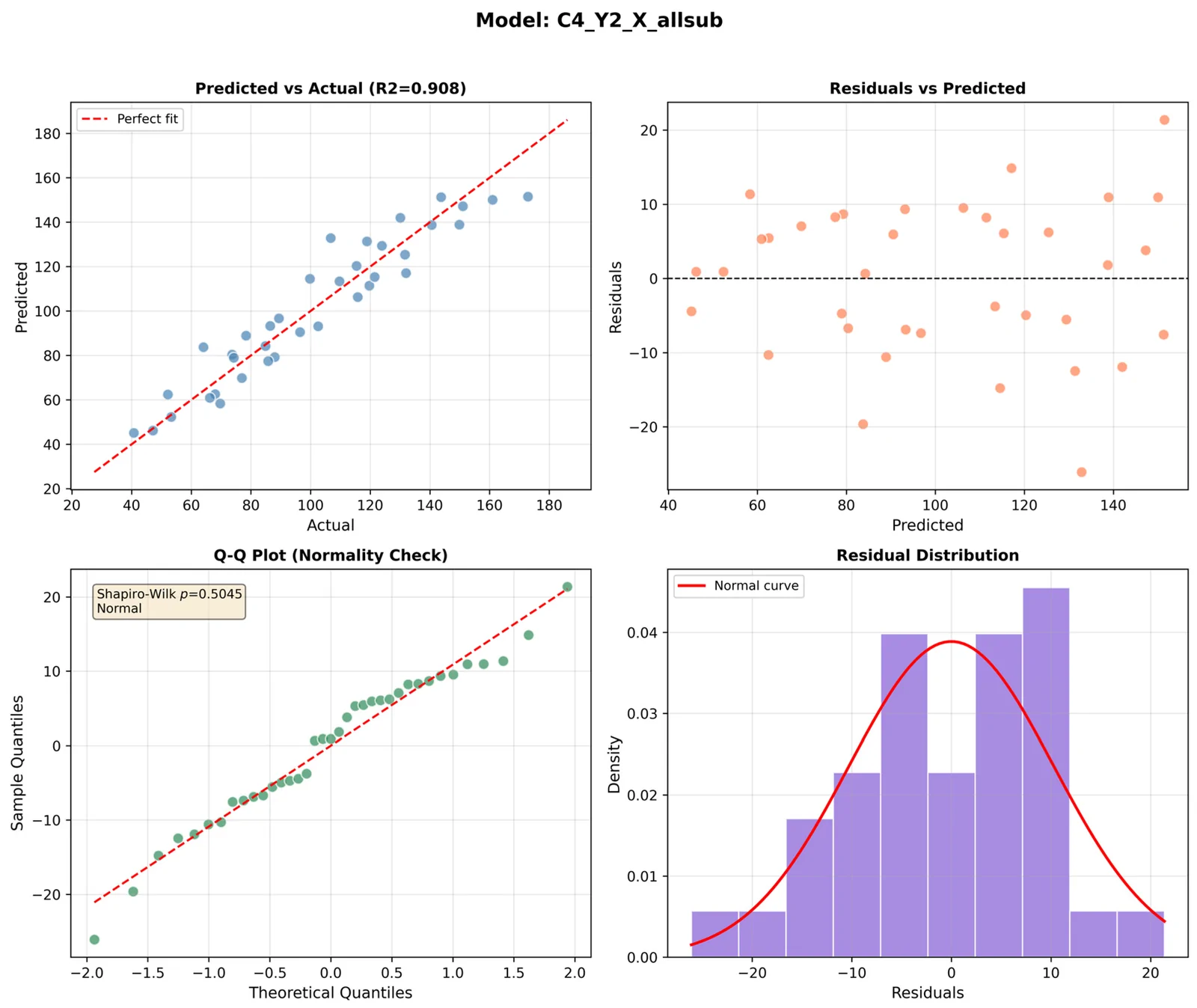

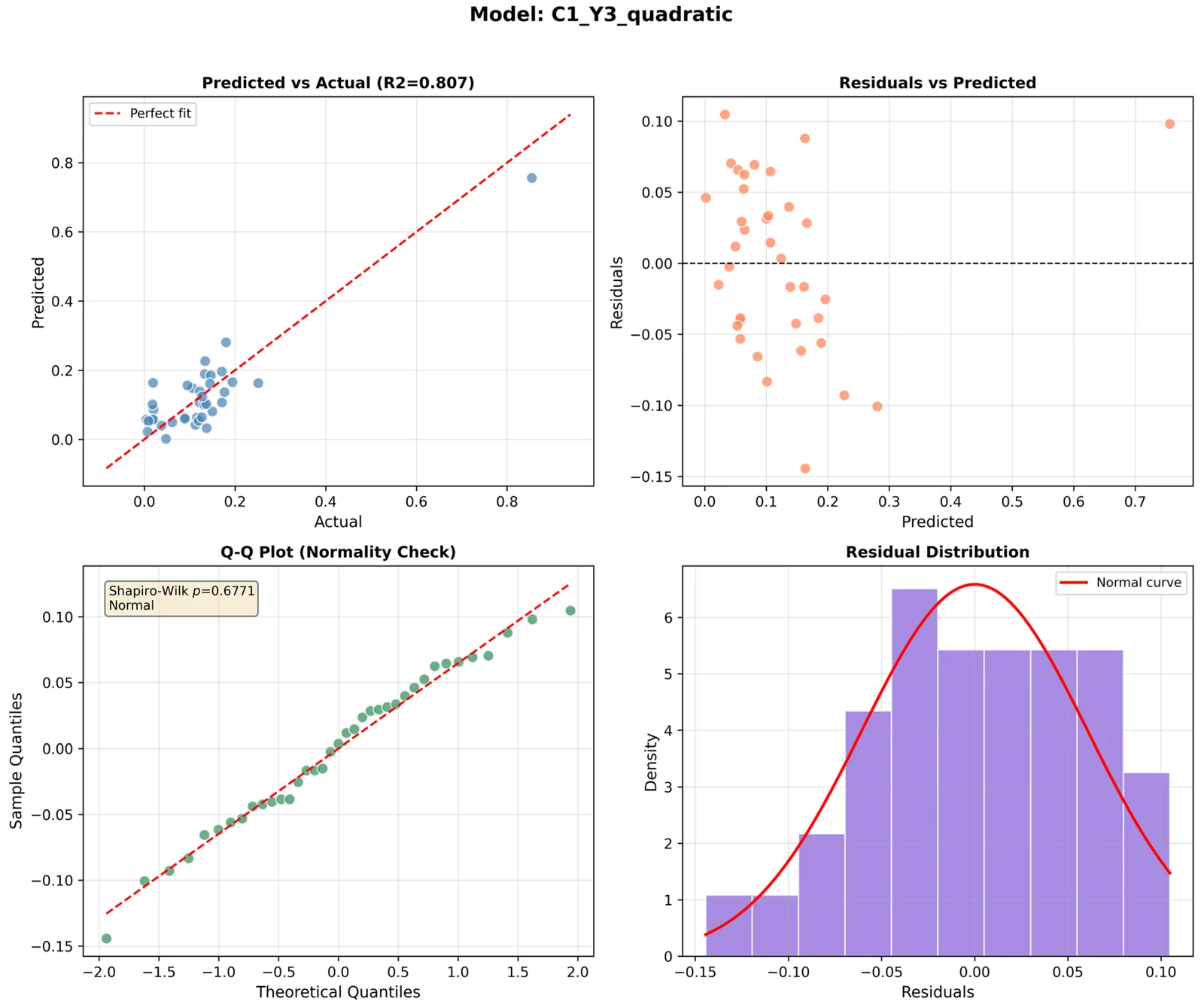
Model diagnostic plots for Y1, Y2, and Y3, arranged from top to bottom. Each four-panel set includes predicted versus actual values with the red dashed 1:1 line, residuals versus predicted values, a Q–Q plot for residual normality, and a residual histogram with a fitted normal curve.

**Figure 6:**
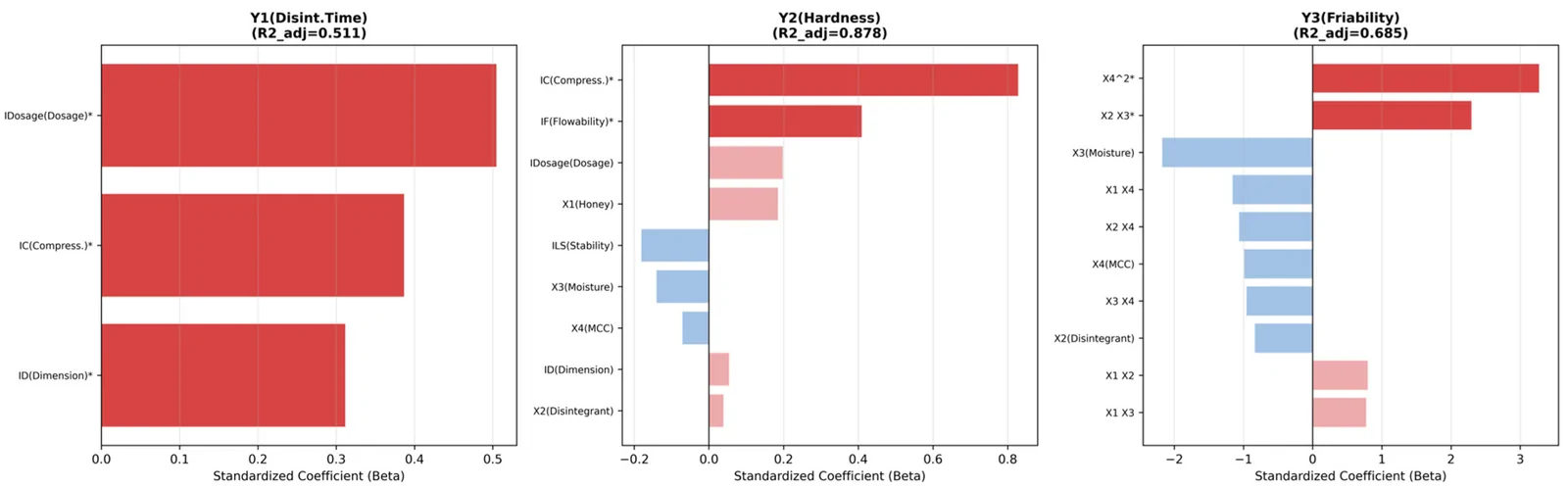
Feature importance plots for Y1, Y2, and Y3 based on standardized coefficients. Bar length shows effect magnitude, red bars indicate positive effects, blue bars indicate negative effects, and adjusted R^2^ values are shown above each panel. * indicates that the standardized regression coefficient for this variable is statistically significant (*p* < 0.05).

Following data processing and analysis, a comparison of all models was carried out, and the optimal prediction model for CQA—with the most favourable parameter values—was selected, as shown in Figure 5. The direct interrelationships between the three factors were also analysed, as shown in Figure 6.

Figure 5 shows the fit and residual diagnostic results for the three CQA optimal simplified models. Overall, the residuals from all three models are randomly distributed around 0. The Q-Q plots and residual histograms indicate that the residuals are approximately normally distributed, with no obvious systematic bias observed, suggesting that the model diagnostic results are generally acceptable. In particular, the Y1 disintegration time model has relatively limited fitting capability, indicating that whilst the model reflects the main trends in disintegration time, the distribution of predicted points is rather scattered, and the accuracy of individual point predictions is poor. The Y2 hardness model performed best, with predicted values closely matching measured values and a relatively stable residual distribution, indicating good explanatory power. The Y3 friability model also yields a good fit; however, the samples are mainly concentrated in the low-friability range, with few high-value samples. Consequently, the predicted results in the high-friability range should be interpreted with caution. Overall, these models can serve as a basis for subsequent key factor screening, path analysis and the construction of design space.

Figure 6 shows the standardised regression coefficients in the optimal models for each CQA. For Y1, the main influencing factors are the SeDeM indicators: IDosage, IC, and ID, each of which has a positive effect. This suggests that the SeDeM indicators may play a mediating role between the formulation factors and disintegration behaviour. About Y2 hardness, IC and IF are the most significant positive factors, indicating that the better the compressibility and flowability, the higher the tablet hardness. The model includes both CPPs and SeDeM parameters, indicating that formulation variables and particle properties jointly govern hardness. Among these, X3, X4, and ILS had negative effects, suggesting that moisture content, the MCC proportion, and stability-related indicators may adversely affect hardness. For Y3 friability, the main influencing factors are the second-order terms and interaction terms of the CPPs, indicating that friability exhibits a distinct non-linear characteristic and cannot be explained by a single main effect alone. To summarize, the results indicate that the SeDeM metric provides a good explanation of Y1 and Y2, whilst the non-linear and interaction effects of the CPPs have a greater influence on Y3. This also supports the need to simultaneously establish the CPPs → CQAs, CPPs → SeDeM, SeDeM → CQAs, and joint models.

### 3.5. Testing the Mediating Effect

**Figure 7:**
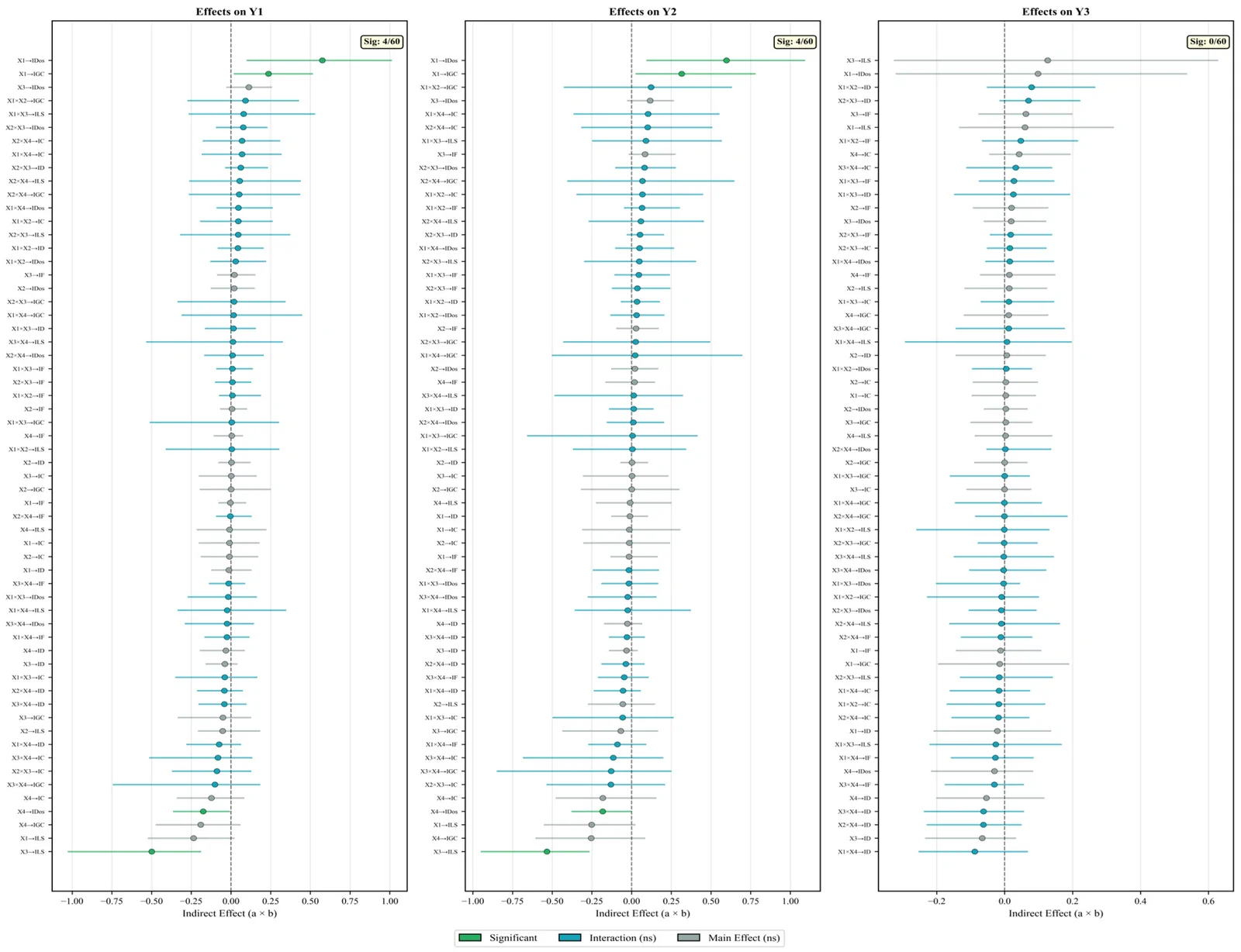
Forest plot of all 180 indirect effects with bootstrap 95% confidence intervals. Points represent estimated indirect effects, horizontal lines indicate 95% CIs, and the vertical dashed line marks zero effect. Green effects are significant, while blue and gray effects indicate nonsignificant interaction and main effects, respectively.

To further investigate the specific mediating effect between these three variables, the results of the Bootstrap model analysis are presented in Table 12 and Figure 7.

Analysis of the mediating effects revealed that, of the total 180 candidate pathways, eight were statistically significant. These significant pathways were all concentrated among the main effect pathways and exhibited relatively large indirect effects, whilst no significant mediating effects were found among the interaction effect pathways. This suggests that the SeDeM metric does not play a widespread role in the transmission process between all prescribing variables and CQAs, but rather exerts a distinctly selective mediating effect. Consequently, in this model, SeDeM is better suited to explaining changes in powder properties caused by variations in individual factors and their impact on CQAs, whilst interaction effects still need to be assessed separately through direct modelling or experimental design. In terms of dimensions, the significant mediating effects were primarily concentrated in the three indicators: IDos, ILS and IGC. IDos is the most significant positive mediating variable, contributing to four significant pathways, indicating that powder properties related to dosage suitability or filling consistency are key mediators influencing Y1 and Y2. It is worth noting that ID, IC, and IF did not exhibit significant mediating effects. This does not imply that these indicators are unimportant; rather, it suggests that, within the current prescription design space, they may influence CQAs more via direct pathways or that their effects have been partially absorbed by composite indicators such as IDos, ILS and IGC. Consequently, these indicators should still be monitored as parameters for powder characterisation, but should not be used as primary mediating explanatory variables.

This analysis should be viewed as exploratory and hypothesis-generating rather than confirmatory. Multiple testing may cause some nominally significant routes to appear by accident. As a result, the mediation results were not employed as the exclusive source of evidence for causal interpretation or formulation decision-making; rather, they were used to indicate possible mechanistic linkages for further research. Therefore, additional comparative analysis using machine learning models is necessary to examine interactive effects and non-linear mechanisms.

### 3.6. Analysis of ML Modelling Results

**Figure 8:**
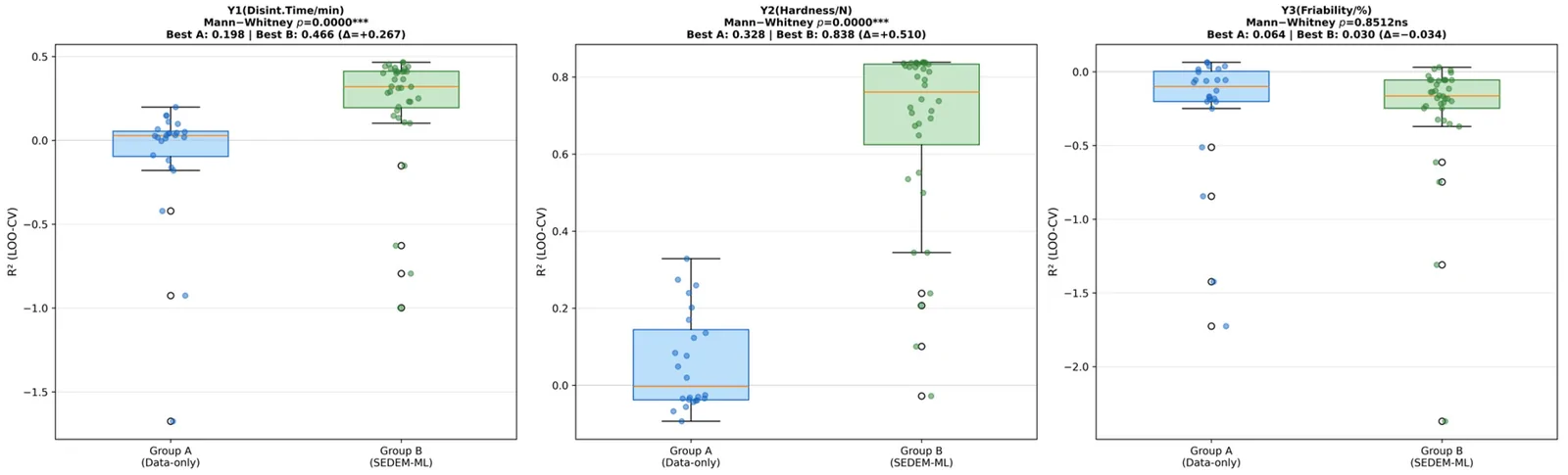
Innovation Proof Data-only (Group A) vs. SeDeM-ML (Group B); Blue represents Group A (Data-only), whereas green represents Group B (SEDEM-ML). Boxplots show cross-validated R^2^ distributions; points indicate individual runs. Mann–Whitney results and best R^2^ values are reported above each panel. *** means *p* < 0.001.

**Figure 9:**
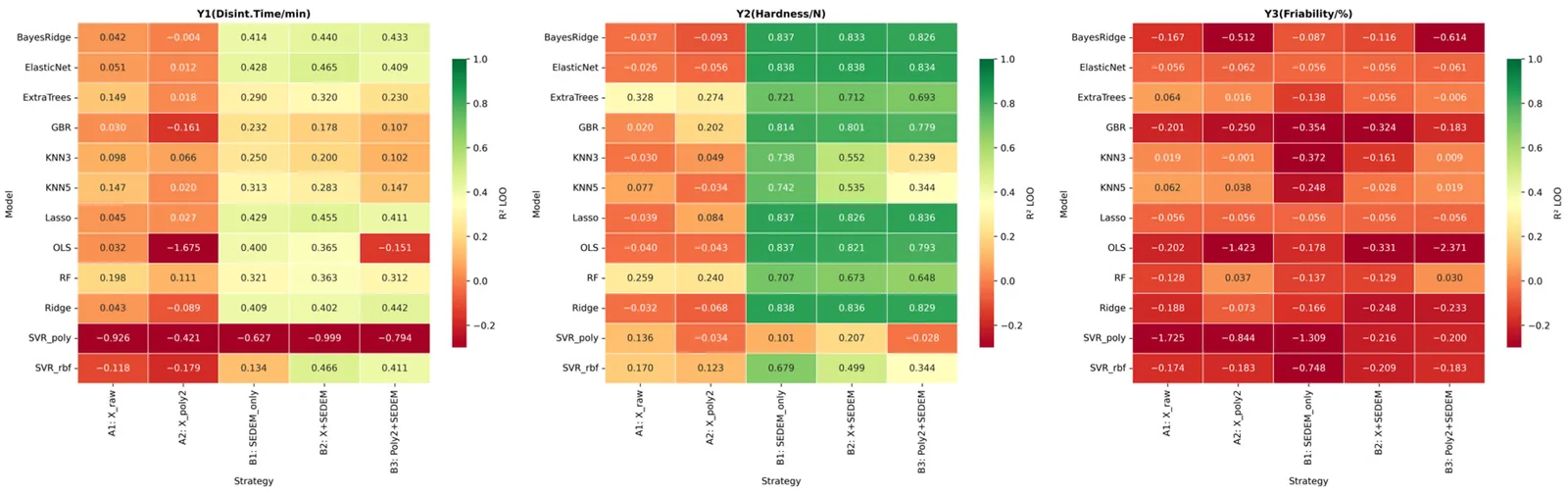
R^2^LOO Heatmap Group A vs. Group B; cell colors and values indicate R^2^LOO, with greener colors representing better performance.

In contrast to the analysis of mediating effects in Section 3.5 regarding interpretability, this section focuses on a systematic comparison of 180 model combinations comprising 3 CQAs, 5 feature strategies and 12 algorithm categories. The selection prioritises models with higher R2_LOO, followed by sufficiently small RMSE_LOO and MAE_LOO, and finally by a smaller Gap to identify the top 10 models for each quality attribute (see Table 13). We then verified whether the SeDeM fusion could yield practical gains in predictive modelling (Figure 8) and evaluated differences in how each model represented individual parameters across the various linear and non-linear relationships analysed in the previous stage (Figure 9).

The results show that the SeDeM-ML hybrid model demonstrates significant advantages for both Y1 and Y2: the R2_LOO for Y1 improved from 0.198 for the pure data model to 0.466, an increase of 0.267; the improvement in hardness for Y2 was most pronounced, rising from 0.328 to 0.838, an increase of 0.510. This indicates that, following the introduction of SeDeM features, the model not only fits the data better but also demonstrates stronger generalisation capabilities, with its predictive scope significantly expanded. In the box plots, the overall distribution for Group B is higher than that for Group A; in particular, several SeDeM fusion strategies in Y2 maintain a high R2, indicating that this improvement is not the result of a single model by chance, but rather an overall advantage arising from the integration of feature mechanism information. The heatmap further shows that, for Y1 and the B2 strategy (i.e., X + SeDeM), the overall performance was the best, with SVR-rbf, ElasticNet, Lasso and BayesRidge all achieving high R2 values, indicating that the decomposition behaviour involves both a certain degree of non-linearity and is consistently supported by the SeDeM mechanism. For Y2, the advantages of SeDeM features were more consistent, with B1, B2 and B3 achieving high R2 values across various models. In particular, regularised linear models such as BayesRidge, ElasticNet, Lasso and Ridge performed exceptionally well, suggesting a strong, interpretable and approximately linear relationship between hardness and the SeDeM metric. By contrast, purely non-linear models such as SVR-poly, partial KNN, and tree-based models exhibited unstable performance on certain CQA datasets and, in some cases, even produced negative R2 values, indicating that complex models are not necessarily superior to simple ones; the key lies in whether effective mechanism features have been incorporated. In the Y3 friability analysis, the overall R2 values for both Group A and Group B were low, indicating that the current set of features has limited predictive power. Overall, this stage has demonstrated that SeDeM fusion does not merely provide a mechanistic explanation; its primary value lies in the fact that, through large-scale model comparisons, it has been established that fusion not only improves the predictive accuracy of Y1 and Y2, but also helps to identify more robust and interpretable model structures, thereby providing a basis for the subsequent development of reliable CQA prediction frameworks and prescription optimisation models.

### 3.7. Construction of the Design Space

**Figure 10:**
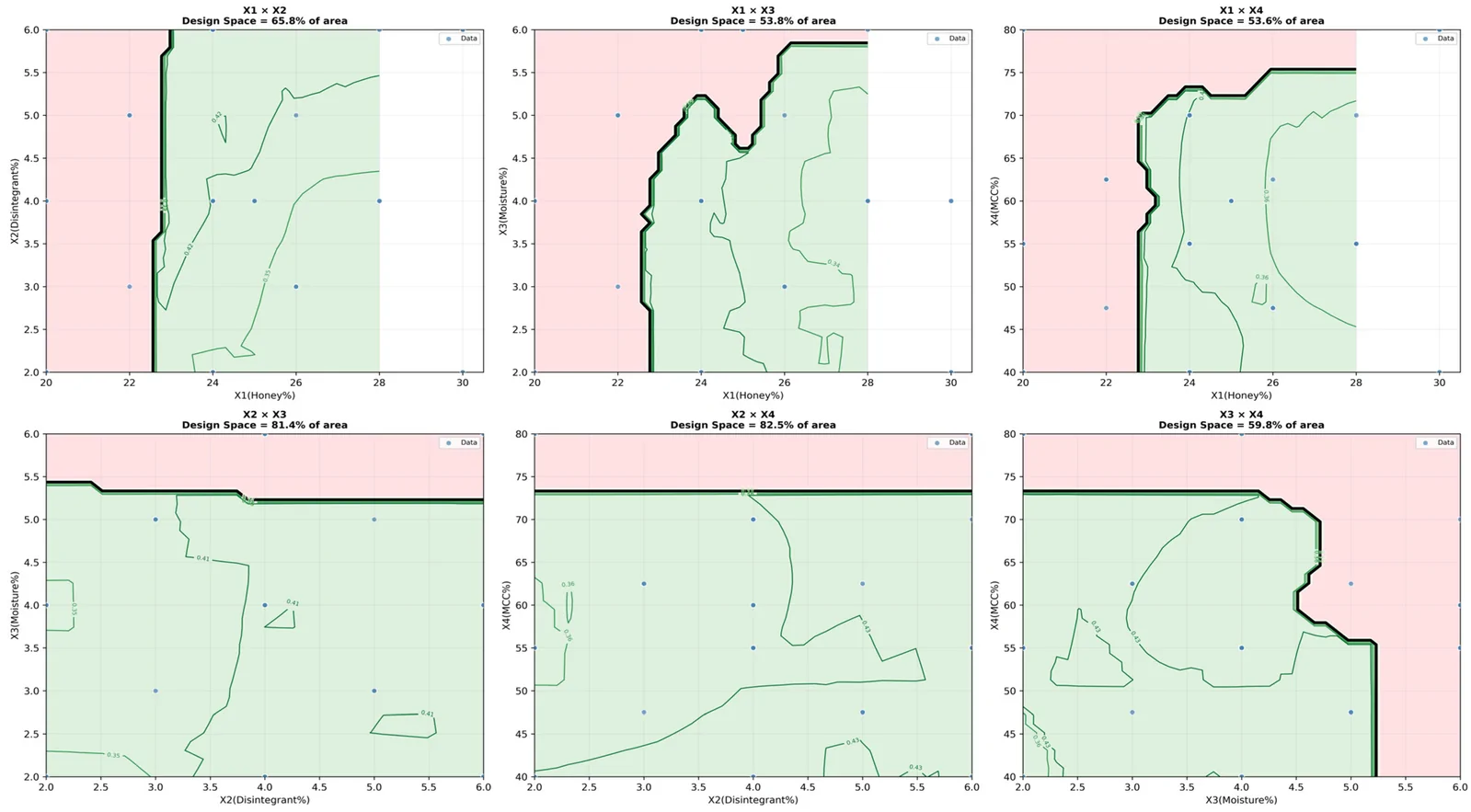
Design Space Map (2D Projections). Green = ALL CQAs pass. Red = at least one CQA fails. The thick black line marks the design-space boundary, and blue points represent experimental runs. Percentages show the acceptable area in each projection. The map defines an acceptable region, not a single optimum.

**Figure 11:**
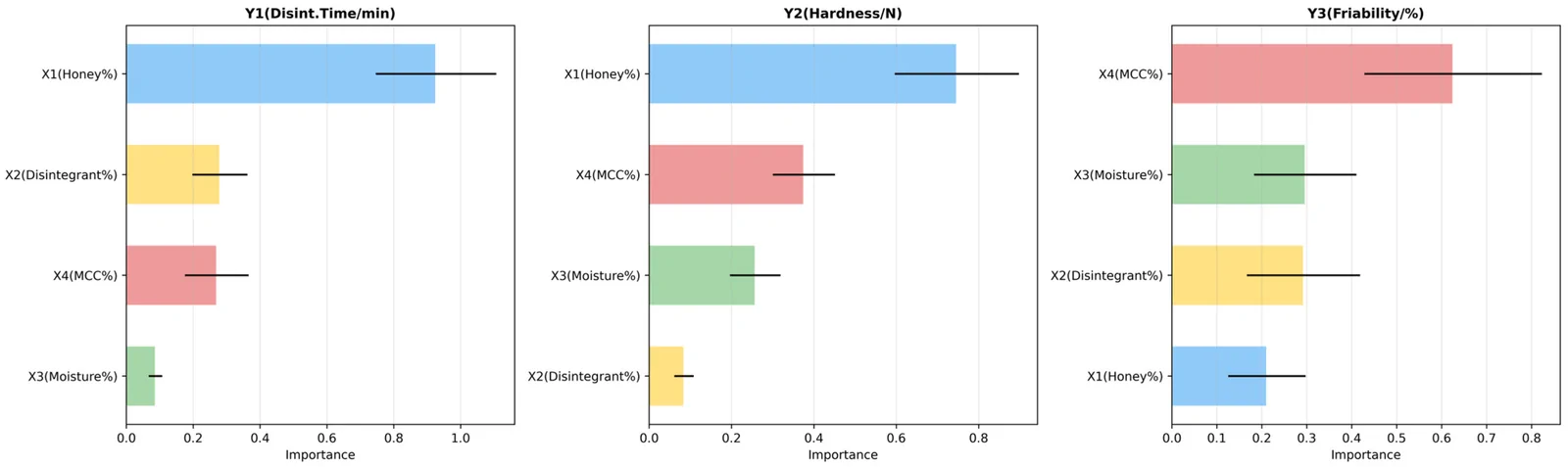
Permutation feature importance of the four formulation factors for each CQA. Bar length indicates influence on model prediction; higher values mean greater importance, and black error bars show variability.

Based on the coverage of the TriAD experimental space, this stage involves a four-dimensional grid scan of 50,625 points, building upon the superior ML model from Section 3.6. The aim is not to output a single optimal formulation, but rather to identify the region of X combinations that satisfies all CQA specifications and yields favourable SeDeM sub-index values (as shown in Figure 10) whilst simultaneously identifying key X factors (Figure 11). This method combines machine-learning predictions, SeDeM feature constraints, and QbD design space principles, thereby enhancing CQA prediction and region identification capabilities and improving the interpretability of formulation changes.

As shown by the aforementioned findings, natural carbohydrate-based materials can significantly influence powder processability and tablet quality attributes. Honey, as a complex natural material rich in carbohydrates, played an important role in modulating the properties of both the powder and the final tablets. This observation is consistent with previous reports showing that chemically modified acorn starch can alter the functional characteristics and sustained-release behavior of monolithic tablets [59]. These findings highlight the importance of thorough material characterization and rational excipient selection when natural carbohydrate-rich ingredients are incorporated into tablet formulations. On this basis, in the formulation design of the present product, the key variable X1 may be fixed according to the inter-batch variability of the Chinese herbal medicine intermediate extract. The resulting formulation variability may then be mitigated by adjusting the non-key variables X2 and X3 within the established design space while appropriately controlling X4. This strategy can improve the consistency of the final preparation and provide practical guidance for industrial-scale production.

### 3.8. Validation Experiment

Three model-predicted formulations were selected; based on the specific constraints of the X variables in the formulations, preparations were prepared and their parameters determined to evaluate the reliability of the model predictions and the validity of the design space. The results are shown in Table 14. 

The results showed that the prediction errors at all three validation points were less than 5 percent, indicating that the model’s predictions were reliable and that all CQAs met their specifications, thereby validating the design space. This further demonstrates the degree of dependability of the SeDeM-ML fusion technology. However, there are still certain restrictions and possible difficulties during the industrialization process in the future. Since honey is a natural substance, batch-to-batch variations in its moisture content, sugar composition, viscosity, and trace elements may have an impact on the particle characteristics and important tablet quality parameters. Granulation effects, tabletting conditions, and equipment variances may introduce additional discrepancies when moving from laboratory batches to industrial production. Second, clear model validation, traceability, and ongoing verification are necessary to get regulatory bodies to acknowledge the machine learning-assisted design space. Thus, additional verification of prescription ratios, honey suppliers, and pilot batches is required to confirm the robustness and generalizability of the suggested methodology.

## 4. Conclusions

In this study, using Yangxin Dawa Yimixike tablets as a model drug, the SeDeM expert system was applied for the first time to a system of honey-containing traditional Chinese medicine tablets. The main conclusions are as follows: the SeDeM system can effectively characterise the direct compression properties of honey-containing traditional Chinese medicine powders. Honey processing is the primary influencing factor, as it affects the various sub-indices of SeDeM and further influences the CQA. This represents the first systematic characterisation of honey processing in tablet powder science. This study established a design scheme based on preliminary screening of excipients, identification of the FFD feasible domain, and a novel TriAD experimental design. In terms of data analysis, various models were thoroughly explored to investigate and demonstrate the interrelationships among formulation factors, SeDeM characteristics, and tablet quality, thereby revealing the significant mediating roles these factors play across different pathways. A multidimensional design space was constructed based on the SeDeM-ML fusion model, and validation results confirmed the model’s reliability across three batches. However, the proposed design space remains inherently dependent on the available experimental data and the assumptions of the predictive model, and uncertainty may be greater near the design-space boundaries. Therefore, the current design space should be interpreted as a model-informed decision-support region rather than an absolute operating range. Recent studies have shown that uncertainty-aware modeling can further improve the trustworthiness of machine-learning-based decision-support systems [60]. Since formal uncertainty quantification or calibration was not included in the present study, this should be recognized as a limitation, and future work should incorporate uncertainty characterization to better define robust formulation regions and strengthen the reliability of ML-assisted QbD decision-making.

Integrating the triad framework comprising SeDeM, ML, and DS provides a new paradigm for QbD research on solid-dose formulations of traditional Chinese medicine. Through IGC evaluation and intermediate modelling, the optimal space is determined; once the interactions between factors have been clarified, the consistency of the finished product can be controlled by regulating multi-factor excipient variables or by controlling intermediate variables. At the same time, the feasible space is interpretable and controllable, exhibiting excellent spatial properties, thereby reducing the need for additional experimental validation, improving the success rate following pilot-scale scale-up, and accelerating industrial translation.

In conclusion, this study developed and verified a framework for the creation of tablets containing honey for traditional Chinese medicine. The findings show that honey-related characteristics, especially its viscosity and hygroscopicity, as well as the adhesive and plasticizing effects brought about by the combined action of soluble sugars and residual moisture, are the main factors influencing the experimental results. These variables also have an impact on the granules’ cohesiveness, bulk density, compressibility, and post-compression pore structure, which eventually result in modifications to the parameters of tablet formulation. As a result, this framework can be used as a guide for the formulation of highly viscous and hygroscopic intermediates, such as those with a high sugar content, semi-solid viscous materials, or hygroscopic extracts, in addition to systems that contain honey. This approach can differentiate between critical variables that need strict control and non-critical variables that may fluctuate within a specific range without significantly affecting quality for traditional Chinese medicine intermediates with batch-to-batch variations in solid content, viscosity, moisture content, and composition. This provides a basis for establishing acceptance limits, enhancing formulation robustness, and lowering batch-to-batch variability. It should be emphasized that the transferable aspect of this study is the development strategy and methodological framework centered on material-specific screening, quality attribute assessment, and design-space construction, rather than the specific formulation parameters. Because material properties and formulation requirements vary across products, the ranges of relevant factors, model parameters, and design-space boundaries should be re-established for each new intermediate and formulation system.

## Figures and Tables

**Table 1:** Five-dimensional parameters of the SeDeM system and their conversion settings.

SeDeM key parameter					
Dimensions					
Symbol	Parameter	Unit	Limit	Equation	Factor
Da	Bulk density	g/mL	0–1.0	Da = P/Va	10v
Dc	Tapped density	g/mL	0–1.0	Dc = P/Vc	10v
Compressibility					
Symbol	Parameter	Unit	Limit	Equation	Factor
Ie	Interparticle porosity	(-)	0–1.2	Ie = (Dc − Da)/(Dc × Da)	v/1.2
IC	Carr index	%	0–50.0	IC = (Dc − Da)/Dc × 100	v/5
Icd	Cohesion index	N	0–200.0	Experimental	v/20
Flowability					
Symbol	Parameter	Unit	Limit	Equation	Factor
IH	Hausner ratio	(-)	1–1.5	IH = Dc/Da	10–10v/0.5
α	Angle of repose	°	0–50.0	Experimental	10–v/5
t″	Powder flow	S	0–20.0	Experimental	10–v/2
Lubricity/Stability					
Symbol	Parameter	Unit	Limit	Equation	Factor
%HR	Loss on drying	%	0–10.0	Experimental	10–v
%H	Hygroscopicity	%	0–20.0	Experimental	10–v/2
Dosage					
Symbol	Parameter	Unit	Limit	Equation	Factor
%Pf	Particles < 50 μm	%	0–50.0	Experimental	10–v/5
Iθ	Homogeneity index	(-)	0–0.02	Experimental	500v

**Table 2:** Formulas of Statistical Models Used to Evaluate the Relationships among CPPs, SeDeM Descriptors, and Tablet CQAs.

Model Type	Formula	Response	Purpose	Interpretation
Main-effect model	R = β0 + ΣβiXi + ε	IGC, five SeDeM sub-indices, Y1, Y2, Y3	Estimate the basic effects of CPP factors on each response.	βi indicates the direction and magnitude of the effect of Xi on the response.
Reduced quadratic model	R = β0 + ΣβiXi + ΣβiiXi^2^ + ΣβijXiXj + ε	IGC, SeDeM sub-indices, Y1, Y2, Y3	Capture significant curvature and two-factor interaction effects.	Xi^2^ describes non-linear curvature; XiXj describes interaction between two factors.
Path-a model	Mk = α0 + ΣαiXi + ε	IGC or SeDeM sub-index	Assess whether CPPs affect particle properties or SeDeM indices.	A significant αi suggests that Xi may influence downstream CQAs through Mk.
Path-b model	Yj = γ0 + ΣγkMk + ε	Y1, Y2, Y3	Assess whether SeDeM indices explain tablet CQAs.	A significant γk indicates that Mk is associated with the corresponding CQA.
Combined model	Yj = δ0 + ΣδiXi + ΣθkMk + ε	Y1, Y2, Y3	Evaluate direct effects of CPPs and potential mediation by SeDeM indices.	If δi decreases after adding Mk and θk is significant, Mk may show partial mediation.
LOOCV evaluation	R^2^LOOCV = 1 − Σ(yi – ŷ − i)^2^/Σ(yi − ȳ)^2^	All modeled responses	Evaluate prediction robustness using all 37 data points.	Higher R^2^LOOCV and lower RMSECV indicate better predictive performance.

**Table 3:** Summary of Variable Definitions, Mediation Paths, and Model Specifications Used in the Statistical Analysis.

Section	Item	Formula	Description	Interpretation
Variable setting	Independent variables	X1–X4	Formulation parameters	Used as predictors to explain SeDeM indices and tablet quality attributes.
Variable setting	Interaction terms	X1 × X2, X1 × X3, X1 × X4, X2 × X3, X2 × X4, X3 × X4	Two-way interaction effects	Used to describe possible synergistic or antagonistic effects among formulation parameters.
Variable setting	Mediators	ID, IC, IF, ILS, IDos, IGC	SeDeM dimensions and global index SeDeM	Tested as potential mediators between formulation parameters and tablet CQAs.
Variable setting	Dependent variables	Y1–Y3	Tablet quality attributes	Used as final responses to evaluate tablet performance.
Path definition	Path a	M = α0 + ΣαiXi + ΣαijXiXj + εM	X → M	Effect of formulation parameters on SeDeM indices
Path definition	Path b	Y = β0 + ΣβiXi + ΣβijXiXj + θM + εY	M → Y|X	Effect of SeDeM index on tablet quality after controlling formulation parameters
Path definition	Path c	Y = γ0 + ΣγiXi + ΣγijXiXj + εY	X → Y	Total effect without mediator
Path definition	Path c′	Y = β0 + ΣβiXi + ΣβijXiXj + θM + εY	X → Y|M	Direct effect after controlling for the mediator
Path definition	Indirect effect	a × b	Mediated effect	Effect transmitted through SeDeM index
Model feature	Multivariate control	Control X1–X4 and interactions	Each X effect is estimated while controlling for other X variables.	Used to estimate the unique contribution of each formulation parameter.
Model feature	Interaction effects	Six two-way interaction terms	X1 × X2, X1 × X3, X1 × X4, X2 × X3, X2 × X4, X3 × X4	Used to capture possible synergistic or antagonistic effects.
Model feature	Multiple mediators	ID, IC, IF, ILS, IDos, IGC	Six SeDeM indicators are tested as potential mediators.	Used to identify which SeDeM dimension may participate in the transmission process.
Model feature	Bootstrap inference	5000 bootstrap samples	Bootstrap 95% confidence interval for a × b	Used to test indirect effects without relying on normality assumption.
Model feature	Model parsimony	Reduced model preferred	Avoid unnecessary predictors under limited sample size.	Used to reduce overfitting and improve interpretability.
Mediation criterion	Significant path a	X significantly predicts M	*p* < 0.05 or CI excludes 0	Formulation parameter is associated with the SeDeM index.
Mediation criterion	Significant path b	M significantly predicts Y|X	*p* < 0.05 or CI excludes 0	SeDeM index is associated with tablet quality after controlling formulation factors.
Mediation criterion	Significant indirect effect	a × b	Bootstrap 95% CI does not include 0	Supports a potential mediation effect.
Mediation criterion	Partial mediation	a × b significant, and c′ remains significant	Both indirect and direct effects exist.	The SeDeM index may partially convey the effects of formulation parameters.
Mediation criterion	Full mediation	a × b significant and c′ not significant	Indirect effect remains, direct effect weakens to non-significance.	The SeDeM index may largely explain the observed X–Y relationship.

**Table 4:** Feature strategies used in machine learning modeling.

Group	Feature Set ID	Feature Set Name	Feature Composition	Methodological Purpose
Group A: Pure data-driven	A1	X_raw	Four original formulation or process factors	Baseline model for evaluating direct predictive ability of original variables
	A2	X_poly2	Original factors + quadratic terms + two-way interactions	To capture nonlinear and interaction effects
Group B: SeDeM-fused	B1	SeDeM_only	Five SeDeM sub-indices	To evaluate the independent predictive ability of SeDeM indices
	B2	X + SeDeM	Original factors + five SeDeM sub-indices	To assess the benefit of combining formulation variables with powder indices
	B3	X_poly2 + SeDeM	Polynomial features + five SeDeM sub-indices	To construct a comprehensive feature set combining nonlinearity and pharmaceutical knowledge

**Table 5:** Summary of Candidate Statistical and Machine-Learning Models Evaluated for Predicting SeDeM Indices and Tablet Critical Quality Attributes.

Model	Model Category	Key Idea	Reason for Inclusion	Key References
OLS	Linear regression	Fits a linear relationship by minimizing residual sum of squares	Provides a baseline linear reference	[43]
Ridge	Regularized linear model	Adds L2 regularization to linear regression	Suitable for multicollinearity, especially in polynomial features	[44]
Lasso	Regularized linear model	Adds L1 regularization to linear regression	Can shrink some coefficients to zero for feature selection	[45]
ElasticNet	Regularized linear model	Combines L1 and L2 regularization	Balances coefficient shrinkage and feature selection	[46]
Bayesian Ridge	Bayesian linear model	Estimates regression parameters under a Bayesian framework	Suitable for limited sample sizes and stable regularization	[47]
KNN3	Local learning model	Predicts based on the three nearest samples	Evaluates the role of local similarity among formulations	[48]
KNN5	Local learning model	Predicts based on the five nearest samples	Provides smoother prediction than KNN3 and may reduce local noise	[48]
SVR_rbf	Kernel-based model	Uses an RBF kernel for nonlinear regression	Suitable for complex nonlinear relationships	[49]
SVR_poly	Kernel-based model	Uses a polynomial kernel for nonlinear regression	Corresponds to polynomial features and captures interactions or curved relationships	[49]
RF	Tree ensemble model	Averages predictions from multiple decision trees	Handles nonlinearity and interactions with robustness to noise	[50]
ExtraTrees	Tree ensemble model	Constructs tree ensembles with more randomized splits	May reduce variance and is suitable for structured tabular data	[51]
GBR	Boosting tree model	Sequentially fits residuals from previous learners	Often shows strong predictive power for complex nonlinear patterns	[52]

**Table 6:** Key methods and formulas for design space construction.

Method	Purpose	Formula	Explanation	References
Specification definition	To define acceptance criteria for CQAs and SeDeM indices.	Y_i ∈ Spec_i, i = 1, 2, 3 S_k ≥ T_k, k = 1, …, K	Y_i represents predicted or measured CQA. Spec_i is the predefined CQA specification. S_k is the SeDeM sub-index, and T_k is its threshold.	ICH Q8(R2)
Grid-based mapping with ML prediction	To evaluate formulation combinations in the 4D input space without exhaustive experiments.	x = (X1, X2, X3, X4) X_i,min ≤ X_i ≤ X_i,max Ŷ_j(x) = f_j(x), j = 1, 2, 3	Each grid point x represents one formulation combination. f_j is the optimal ML model used to predict the j-th CQA.	[18]
Design space identification and 2D projection	To identify the feasible multidimensional region rather than a single optimum.	D_space = {x ∈ X: Ŷ1 ∈ Spec_1, Ŷ2 ∈ Spec_2, Ŷ3 ∈ Spec_3, S_k ≥ T_k} P_ij(D_space) = {(X_i, X_j): ∃ remaining X values such that x ∈ D_space}	A point belongs to the design space only when all CQA and SeDeM constraints are satisfied. 2D projections are used to visualize the 4D feasible region.	[54]
Constraint bottleneck analysis	To determine which CQA or SeDeM constraint limits the design space.	Failure rate_j = N_fail,j/N_total For upper-limit constraint: M_j(x) = (U_j − Ŷ_j)/(U_j − L_j) For range constraint: M_j(x) = min[(Ŷ_j − L_j), (U_j − Ŷ_j)]/(U_j − L_j)	The failure rate quantifies how frequently each constraint is violated. M_j describes the normalized distance to a constraint boundary.	[55]
Desirability-based optimization	To select a recommended formulation within the feasible design space.	D = (d1 × d2 × … × dm)^(1/m) or weighted form: D = (Π d_i^w_i)^(1/Σw_i)	d_i ranges from 0 to 1 and represents the desirability of each response. A higher D indicates better overall performance.	[56]
Robustness analysis	To avoid selecting points close to failure boundaries.	Distance-based robustness: R_dist(x) = min_j M_j(x) Perturbation-based robustness: R_prob = N_pass/N_MC	R_dist measures the minimum distance to the nearest constraint boundary. R_prob estimates the probability that perturbed points remain acceptable.	[57]

**Table 7:** Comparison of basic SeDeM parameters and IGC among different excipient combinations.

Sample	r_Da	r_Dc	r_Ie	r_IC	r_Icd	r_IH	r_α	r_t″	r_%HR	r_%H	r_%Pf	r_Iθ	IPP	IGC
P1	4.24	5.63	4.85	4.94	4.00	8.36	2.93	6.96	6.97	5.98	3.54	0.78	4.93	4.70
P2	4.25	5.44	4.29	4.38	4.60	8.60	3.04	6.71	6.40	5.53	8.69	0.93	5.24	4.99
P3	4.06	5.06	4.06	3.95	3.35	8.77	2.07	5.94	5.47	5.16	9.00	0.98	4.82	4.59
P4	3.78	4.87	4.93	4.48	3.70	8.56	2.62	5.74	6.23	5.46	9.42	1.48	5.11	4.86
P5	4.24	5.44	4.34	4.41	4.75	8.58	2.52	6.78	6.64	5.84	8.44	1.46	5.29	5.03
P6	3.99	5.26	5.04	4.83	4.45	8.41	2.47	6.57	6.10	5.59	4.44	0.62	4.81	4.58
P7	3.88	5.12	5.20	4.84	3.45	8.40	2.27	6.08	6.74	4.50	8.39	0.95	4.99	4.75
P8	3.92	5.08	4.85	4.57	2.65	8.52	2.03	6.16	6.75	5.74	7.19	0.91	4.86	4.63

**Table 8:** Comparative analysis of IGC and CQA results across different excipient combinations.

Sample	IGC	Disintegration Time (min)	Hardness (N)	Friability (%)
P1	4.70	8.65	90.28	0.06
P2	4.99	5.28	92.89	0.08
P3	4.59	5.80	67.25	0.04
P4	4.86	7.06	82.76	0.17
P5	5.03	6.38	93.60	0.04
P6	4.58	8.60	89.53	0.09
P7	4.75	7.25	69.01	0.04
P8	4.63	7.20	53.70	0.10

**Table 9:** Results of the screening of different disintegrants.

Sample	Major Excipients	Disintegrants	Dosage %	Disintegration Time (min)	Hardness (N)	Friability (%)
P9	MCC 19.9% + Dextrin 16%	PVPP	5	6.38	93.60	0.04
P10	MCC 19.9% + Dextrin 16%	CMS-Na	5	11.60	84.21	0.17
P11	MCC 19.9% + Dextrin 16%	Starch	5	9.46	40.97	0.48

**Table 10:** Comparison of IGC and CQA results for different combinations of excipients.

Sample	ID	IC_dim	IF	ILS	IDosage	IGC	Disintegration Time (min)	Hardness (N)	Friability (%)
S1	4.18	3.62	5.72	5.84	2.23	4.17	5.60	47.15	0.85
S2	4.00	4.05	5.17	5.14	5.34	4.49	8.18	87.98	0.25
S3	3.83	4.51	5.22	5.55	2.79	4.24	6.35	68.01	0.05
S4	3.73	4.49	5.25	5.14	5.07	4.53	8.03	86.43	0.04
S5	3.64	4.67	5.49	5.19	3.67	4.40	3.75	64.09	0.02
S6	3.57	3.89	5.11	4.83	5.36	4.32	6.38	53.27	0.13
S7	3.94	3.59	5.77	5.53	2.07	4.06	2.78	40.73	0.13
S8	4.08	3.97	5.61	4.59	6.11	4.62	6.97	109.64	0.02
S9	3.85	3.90	5.68	4.73	4.24	4.31	6.75	85.77	0.02
S10	3.67	3.97	5.51	4.45	4.23	4.22	6.00	76.94	0.01
S11	3.61	3.79	5.51	4.72	4.46	4.25	5.25	66.23	0.02

**Table 11:** Complete data from the TriADs experiment (X + SeDeM + Y) results.

Sample	X (%)				SeDeM						CQA		
	X1	X2	X3	X4	IGC	ID	IC	IF	ILS	IDos	Y1 (min)	Y2 (N)	Y3 (%)
A1	20.00	4.00	4.00	55.00	4.27	4.15	3.94	5.74	6.00	2.22	5.60	69.71	0.12
A2	28.00	4.00	4.00	55.00	5.04	3.75	5.47	5.69	5.94	5.34	9.52	151.04	0.06
A3	24.00	2.00	4.00	55.00	5.00	3.86	5.98	5.69	6.03	4.10	9.03	160.99	0.01
A4	24.00	6.00	4.00	55.00	4.89	3.94	5.47	5.68	5.76	4.38	7.63	140.57	0.11
A5	24.00	4.00	2.00	55.00	4.99	3.83	5.50	5.49	6.46	4.65	7.92	131.63	0.11
A6	24.00	4.00	6.00	55.00	4.83	3.68	5.92	5.45	4.88	4.83	7.67	130.07	0.12
A7	24.00	4.00	4.00	40.00	4.94	4.16	5.45	5.74	5.43	4.75	7.13	143.74	0.12
A8	24.00	4.00	4.00	70.00	4.82	3.71	5.79	5.28	5.92	4.13	6.58	99.77	0.19
B1	20.00	2.00	4.00	55.00	4.62	4.08	5.53	5.30	5.96	2.82	9.47	102.52	0.13
B2	28.00	6.00	4.00	55.00	4.94	4.01	4.80	5.64	5.56	5.89	7.87	123.88	0.14
B3	20.00	4.00	2.00	55.00	4.54	3.82	5.02	5.12	6.55	3.03	6.25	52.18	0.17
B4	28.00	4.00	6.00	55.00	4.83	4.08	4.97	5.64	4.89	5.54	9.13	106.75	0.12
B5	20.00	4.00	4.00	40.00	4.63	4.23	4.76	5.57	5.65	3.79	5.93	89.37	0.18
B6	28.00	4.00	4.00	70.00	4.95	4.29	4.71	5.77	5.53	5.64	9.58	118.90	0.17
B7	24.00	2.00	2.00	55.00	4.82	3.91	4.76	5.24	6.45	5.05	10.22	73.67	0.18
B8	24.00	6.00	6.00	55.00	4.74	3.83	5.13	5.47	4.90	5.24	7.75	131.98	0.15
B9	24.00	2.00	4.00	40.00	4.96	3.77	5.65	5.28	5.87	5.23	8.02	115.39	0.13
B10	24.00	6.00	4.00	70.00	4.91	3.69	5.49	5.25	5.73	5.41	7.42	119.67	0.15
B11	24.00	4.00	2.00	40.00	4.76	3.83	4.65	5.33	6.46	4.72	8.72	84.89	0.14
B12	24.00	4.00	6.00	70.00	4.55	3.79	4.51	5.50	4.72	5.14	8.17	96.47	0.14
C1	22.00	5.00	3.00	62.50	4.57	3.69	5.26	5.28	5.97	3.33	5.20	78.33	0.02
C2	26.00	3.00	5.00	47.50	4.84	3.76	5.22	5.32	5.24	5.71	9.13	121.45	0.01
C3	22.00	3.00	5.00	62.50	4.60	3.97	5.12	5.49	5.04	4.05	8.10	115.82	0.09
C4	26.00	5.00	3.00	47.50	5.09	4.02	5.83	5.42	5.70	5.49	8.62	172.86	0.09
C5	26.00	3.00	3.00	62.50	5.10	3.95	5.59	5.48	5.94	5.64	10.03	149.86	0.09
C6	22.00	5.00	5.00	47.50	4.60	3.74	4.90	5.11	5.18	5.08	4.50	74.26	0.13

**Table 12:** Results of the Exploratory Mediation Analysis of the Effects of Formulation Parameters on Tablet Quality Attributes Through SeDeM Indices.

X	Type	M	Y	a	a_p	b	b_p	C′	c′_p	a × b	Boot_CI	Conclusion
X1	Main	IDos	Y1	0.827	<0.0001	0.695	0.0142	−0.056	0.8298	0.5751	[0.102, 1.008]	Full mediation
X1	Main	IDos	Y2	0.827	<0.0001	0.722	0.0181	−0.233	0.4117	0.5974	[0.097, 1.087]	Full mediation
X1	Main	IGC	Y1	0.371	0.0254	0.637	<0.0001	0.283	0.0201	0.2362	[0.022, 0.511]	Partial mediation
X1	Main	IGC	Y2	0.371	0.0254	0.851	<0.0001	0.049	0.5777	0.3156	[0.029, 0.776]	Full mediation
X3	Main	ILS	Y1	−0.678	<0.0001	0.736	0.0007	0.394	0.0359	−0.4992	[−1.025, −0.193]	Partial mediation
X3	Main	ILS	Y2	−0.678	<0.0001	0.788	0.0007	0.664	0.0017	−0.534	[−0.946, −0.270]	Partial mediation
X4	Main	IDos	Y1	−0.252	0.0076	0.695	0.0142	0.045	0.7674	−0.1752	[−0.360, −0.011]	Full mediation
X4	Main	IDos	Y2	−0.252	0.0076	0.722	0.0181	−0.119	0.4679	−0.182	[−0.374, −0.006]	Full mediation

**Table 13:** Comparative Predictive Performance of the Top 10 Models for Each Tablet Critical Quality Attribute.

CQA	Strategy	Model	n_Feat	R^2^_Train	R^2^_LOO	RMSE_LOO	MAE_LOO	Gap
Y1	B2: X + SeDeM	SVR_rbf	9	0.9968	0.4656	1.2677	0.9653	0.5312
Y1	B2: X + SeDeM	ElasticNet	9	0.6819	0.4652	1.2682	1.0288	0.2167
Y1	B2: X + SeDeM	Lasso	9	0.6707	0.4547	1.2806	1.0272	0.2161
Y1	B3: Poly2 + SeDeM	Ridge	19	0.7982	0.4423	1.2950	1.0389	0.3559
Y1	B2: X + SeDeM	BayesRidge	9	0.6873	0.4403	1.2974	1.0545	0.2470
Y1	B3: Poly2 + SeDeM	BayesRidge	19	0.7140	0.4334	1.3054	1.0503	0.2806
Y1	B1: SeDeM_only	Lasso	5	0.5764	0.4290	1.3104	1.0582	0.1474
Y1	B1: SeDeM_only	ElasticNet	5	0.5809	0.4285	1.3110	1.0650	0.1525
Y1	B1: SeDeM_only	BayesRidge	5	0.5833	0.4141	1.3274	1.0812	0.1692
Y2	B2: X + SeDeM	ElasticNet	9	0.9032	0.8382	13.4465	11.4124	0.0649
Y2	B1: SeDeM_only	Ridge	5	0.8846	0.8382	13.4470	11.0827	0.0464
Y2	B1: SeDeM_only	ElasticNet	5	0.883	0.838	13.463	11.148	0.045
Y2	B1: SeDeM_only	BayesRidge	5	0.8848	0.8373	13.4845	11.1033	0.0475
Y2	B1: SeDeM_only	OLS	5	0.8852	0.8366	13.5125	11.0958	0.0486
Y2	B1: SeDeM_only	Lasso	5	0.8852	0.8366	13.5145	11.0942	0.0486
Y2	B3: Poly2 + SeDeM	Lasso	19	0.9334	0.8358	13.5489	11.2156	0.0976
Y2	B2: X + SeDeM	Ridge	9	0.9067	0.8357	13.5498	11.4736	0.0709
Y2	B3: Poly2 + SeDeM	ElasticNet	19	0.9097	0.8336	13.6371	11.6154	0.0761
Y2	B2: X + SeDeM	BayesRidge	9	0.9061	0.8332	13.6523	11.5501	0.0729
Y2	B2: X + SeDeM	ElasticNet	9	0.9032	0.8382	13.4465	11.4124	0.0649
Y3	A1: X_raw	ExtraTrees	4	0.6572	0.0640	0.1316	0.0719	0.5932
Y3	A1: X_raw	KNN5	4	0.9998	0.0622	0.1317	0.0708	0.9376
Y3	A2: X_poly2	KNN5	14	0.9998	0.0381	0.1334	0.0689	0.9617
Y3	A2: X_poly2	RF	14	0.5837	0.0368	0.1335	0.0690	0.5468
Y3	B3: Poly2 + SeDeM	RF	19	0.6372	0.0304	0.1340	0.0711	0.6067
Y3	B3: Poly2 + SeDeM	KNN5	19	1.0000	0.0194	0.1347	0.0747	0.9806
Y3	A1: X_raw	KNN3	4	0.9998	0.0186	0.1348	0.0771	0.9812
Y3	A2: X_poly2	ExtraTrees	14	0.6456	0.0162	0.1349	0.0731	0.6293
Y3	B3: Poly2 + SeDeM	KNN3	19	1.0000	0.0092	0.1354	0.0756	0.9908
Y3	A2: X_poly2	KNN3	14	0.9998	−0.0009	0.1361	0.0766	1.0007
Y3	A1: X_raw	ExtraTrees	4	0.6572	0.0640	0.1316	0.0719	0.5932

**Table 14:** Comparison of Model-Predicted and Experimentally Observed Tablet Quality.

Project	Verification Points 1	Verification Points 2	Verification Points 3
X1 (%)	23.4	22.5	27.6
X2 (%)	6	3.5	3.1
X3 (%)	4.1	4.8	4.0
X4 (%)	40	65.5	52
Y1Forecast	6.57	7.75	10.44
Y1Actual	6.25	7.97	10.10
Y1Deviation%	4.87%	2.84%	3.26%
Y2Forecast	119.89	98.07	127.07
Y2Actual	115.66	93.88	121.74
Y2Deviation%	3.53%	4.27%	4.19%
Y3Forecast	0.1011%	0.1141%	0.0899%
Y3Actual	0.0986%	0.1191%	0.0867%
Y3Deviation%	2.47%	4.38%	3.56%

## Data Availability

All original data from this work are contained in the article. Readers seeking further details may direct their inquiries to the corresponding author upon reasonable request.

## Supplementary Materials

The following supporting information can be downloaded at: https://www.mdpi.com/article/10.3390/pharmaceutics18081014/s1, SeDeM measurement method; Table S1. Formulation design for screening of major excipients; Table S2. Formulation design for screening of disintegrants; Table S3. Experimental runs and formulation composition of the FFD at different factor levels; Table S4. Integration of Three Classical Design Principles in TriAD; Table S5. Complete experimental matrix for TriAD; Table S6. Evaluation_Criteria.
