# Supplementary material for: QbD-Based Design Space Development for Honey-Containing Traditional Chinese Medicine Tablets Assisted by the SeDeM Expert System and Machine Learning
Source: Pharmaceutics. 2026 Aug 16;18(8):1014. doi: 10.3390/pharmaceutics18081014 (PMC13516143; doi:10.3390/pharmaceutics18081014)
Supplement: Supplementary file 1 [file pharmaceutics-18-01014-s001.zip › pharmaceutics-4469511-supplementary.pdf]

Supplementary Materials:

2.2. SeDeM measurement method

The SeDeM system creates the SeDeM polygon map by joining the radii of each parameter after converting the experimental values of the twelve fundamental parameters into radii in the range of 0–10 based on the sample findings displayed in Table 1. The SeDeM approach additionally incorporates three composite indices: the Parameter Index (IP), the Parameter Profile Index (IPP), and the Good Compressibility Index (IGC), to thoroughly assess the suitability of powders for direct tableting. These indices are computed as follows:

$$IP=\frac{NoP_{\geq 5}}{NoP_t} \quad (1)$$

$$IPP=\frac{\sum r_i}{NoP_t} \quad (2)$$

$$IGC=IPP\times f \quad (3)$$

$$f=\frac{Polygonarea}{Circlearea} \quad (4)$$

Here, $NoP_{\geq 5}$ represents the number of parameters with a radius value of at least 5, NoP_t represents the total number of test parameters.$r_i$represents the radius values of each parameter, and$f$ represents the reliability factor.

2.2.1 Methods for Determining Parameters Related to Dimensions

Bulk density (Da): In accordance with Method 1 of General Rule 0993 of the 2025 Edition of the Chinese Pharmacopoeia, take approximately 100 g of the powder sample to be tested, weigh it accurately, and slowly pour it into a graduated glass measuring cylinder. Avoid compacting the powder; record the apparent volume at the nearest graduation mark, and calculate the bulk density using Equation (5):

$$Da=\frac{M}{V0} \quad (5)$$

In Equation (5), Da is the bulk density determined by the fixed-mass method, in g/ml; M is the mass of the sample under test, in g; and V_0 is the apparent volume of the sample under test, in ml.

Tapped density (Dc): Follow the procedure for bulk density (Method 1). Secure the graduated cylinder, which has been filled with the powder in a loose state, to the stand. Tap it 10, 500 and 1250 times, recording the corresponding volumes V10, V500 and V1250, accurate to the nearest minimum graduation. If the difference between V500 and V1250 is no greater than 2 ml, take V1250 as the tapped volume; if the difference is greater than 2 ml, increase the number of tapping strokes until the difference between two consecutive recorded volumes is no greater than 2 ml. The final tapped volume is used to calculate the tapped density using the following formula:

$$Dc=\frac{m}{V} \quad (6)$$

In Equation (6), Dc is the tapped density determined by the constant-mass method, in g/ml; m is the mass of the sample to be tested, in g; and V is the tapped volume of the sample to be tested, in ml.

2.2.2 Methods for Determining Parameters Related to Dimensional Compressibility

Interparticle porosity (Ie): This parameter was calculated by Da and Dc from Equation

$$Ie=\frac{Dc-Da}{Dc\times Da} \quad (7)$$

Carr index(IC): This parameter was calculated as follows:

$$IC=\frac{Dc-Da}{Dc}\times 100 \quad (8)$$

Cindex (Icd): This parameter was obtained from the hardness (N) of the tablets.

2.2.3 Methods for Determining Flowability-Related Parameters in Dimension

Hausner ratio (IH): Hausner ratio was calculated by Dc and Da using Equation (9):

$$IH=\frac{Dc}{Da} \quad (9)$$

Angle of repose (α): This parameter was tested according to Ph. Eur. in Section 2.9.36 using standard apparatus. Basic methods were used to determine the static angle of repose. This method measured the height (h) and the base (r) of the powder cone, and α was calculated using Equation (10).

$$\tan(\alpha)=\frac{h}{r} \quad (10)$$

Powder flow (t"): This parameter was tested according to Ph. Eur. in Section 2.9.16 using the standard apparatus. Place the powder in the funnel, allow it to pass through the funnel's opening, and record the time it takes to flow through.

2.2.4 Methods for determining parameters related to Lubricity and Stability

Loss on drying (%HR): The test method for this parameter was described in the Ph. Eur. (Section 2.2.32). The sample was dried at 105 ± 2°C until a constant weight was reached.

Hygroscopicity (%H): The hygroscopicity of the particles was assessed by measuring their mass gain over 24 hours under controlled humidity conditions (75 ± 5%); the percentage mass gain was used as a quantitative indicator of hygroscopicity.

2.2.5 Methods for Determining Dosage-Related Parameters in Dimension

Particle size (%Pf): The percentage of fine particles (<75 micrometres) was determined using sieve analysis.

Homogeneity index (Iθ): The parameter was determined by the sieve test in accordance with the Ph. Eur. (Section 2.9.12).The sieve sizes included 1700μm, 1055μm, 1700μm, 505 μm, 256μm, 196μm, 165μm and 50 μm. The percentage of powder retained in each sieve was calculated. The homogeneity index was calculated by Equation (11).

$$I\theta=\frac{Fm}{100+(d_m-d_{m-1}-1)F_{m-1}+(d_{m+1}-d_m)F_{m+1}+((d_m-d_{m-2})F_{m-2}+(d_{m+2}-d_m)F_{m+2}\cdots+(d_m-d_{m-n})F_{m-n}+(d_{m+n}-d_m)F_{m+n}} \quad (11)$$

where Iθ is the homogeneity index.

Fm, percentage of powder in the majority range;

Fm-1 and Fm+1, percentage of powder in the range immediately below/above the majority range;

n, the number of the fraction studied under a series, with respect to the major fraction;

dm, mean diameter of the powder in the major fraction;

dm-1 and dm+1 denote the powder diameter in the fraction immediately below/above the majority range.

Tables

Table S1. Formulation design for screening of major excipients

Sample	Intermediate (%)	Refined honey solids (%)	Major excipient A	Major excipient A (%)	Major excipient B	Major excipient B (%)	Disintegrant	Disintegrant(%)	Glidant	Glidant (%)	Lubricant	Lubricant (%)	Total (%)
P1	35.5	22.1	MCC	35.9	—	0	PVPP	5	SiO_2	0.5	Mg-St	1	100
P2	35.5	22.1	Dextrin	35.9	—	0	PVPP	5	SiO_2	0.5	Mg-St	1	100
P3	35.5	22.1	Starch	35.9	—	0	PVPP	5	SiO_2	0.5	Mg-St	1	100
P4	35.5	22.1	Lactose	35.9	—	0	PVPP	5	SiO_2	0.5	Mg-St	1	100
P5	35.5	22.1	MCC	19.9	Dextrin	16	PVPP	5	SiO_2	0.5	Mg-St	1	100
P6	35.5	22.1	MCC	25.9	Lactose	10	PVPP	5	SiO_2	0.5	Mg-St	1	100
P7	35.5	22.1	Dextrin	25.9	Starch	10	PVPP	5	SiO_2	0.5	Mg-St	1	100
P8	35.5	22.1	MCC	13.9	Dextrin+lactose	22	PVPP	5	SiO_2	0.5	Mg-St	1	100

Note: MCC, microcrystalline cellulose; PVPP, crospovidone; SiO_2, colloidal silicon dioxide; Mg-St, magnesium steara

Table S2. Formulation design for screening of disintegrants

Sample	Intermediate (%)	Refined honey solids (%)	Major excipient A	Major excipient A (%)	Major excipient B	Major excipient B (%)	Disintegrant	Disintegrant (%)	Glidant	Glidant (%)	Lubricant	Lubricant (%)
P9	35.5	22.1	A	As determined from the results of major excipient screening	B	As determined from the results of major excipient screening	PVPP	5	SiO_2	0.5	Mg-St	1
P10	35.5	22.1	A		B		CMS-Na	5	SiO_2	0.5	Mg-St	1
P11	35.5	22.1	A		B		Starch	5	SiO_2	0.5	Mg-St	1

Note: A and B represent the selected major excipient combination obtained from the results of the section; CMS-Na, sodium carboxymethyl starch

Table S3. Experimental runs and formulation composition of the FFD at different factor levels

Run No	Coded level of X1	Coded level of X2	Coded level of X3	Coded level of X4	Refined honey (%)	Disintegrant (%)	Moisture content (%)	MCC ratio (%)
S1	-1	-1	-1	1	20	2	2	80
S2	1	-1	-1	-1	30	2	2	40
S3	-1	1	-1	-1	20	6	2	40
S4	1	1	-1	1	30	6	2	80
S5	-1	-1	1	-1	20	2	4	40
S6	1	-1	1	1	30	2	4	80
S7	-1	1	1	1	20	6	4	80
S8	1	1	1	-1	30	6	4	40
S9	0	0	0	0	25	4	3	60
S10	0	0	0	0	25	4	3	60
S11	0	0	0	0	25	4	3	60

Table S4 Integration of Three Classical Design Principles in TriAD

Design Principle	Core Principle	Contribution to TriAD	Implementation in TriAD
Orthogonal Design	Balanced allocation of factor levels	Stable estimation of main effects and selected interactions reduces confounding in model interpretation.	Factorial core inherited from the previous screening phase, with necessary axial or center points
Uniform Design	Space-filling distribution of experimental points	Improves coverage of the design region and reduces the risk of point clustering	Addition of face-centered and space-filling points to explore the interior region

D-Optimal Design	Maximization of information matrix ∣X'X∣	Improves parameter estimation efficiency and reduces estimation variance under a specified model	Adaptive selection of candidate points according to the D-optimal criterion

Table S5. Complete experimental matrix for TriAD

Group	Component	X_1	X_2	X_3	X_4	Design Principle
1	Axial A	−1	0	0	0	Orthogonal
2	Axial A	+1	0	0	0	Orthogonal
3	Axial A	0	−1	0	0	Orthogonal
4	Axial A	0	+1	0	0	Orthogonal
5	Axial A	0	0	−1	0	Orthogonal
6	Axial A	0	0	+1	0	Orthogonal
7	Axial A	0	0	0	−1	Orthogonal
8	Axial A	0	0	0	+1	Orthogonal
9	Face B	+1	+1	0	0	Uniform
10	Face B	+1	−1	0	0	Uniform
11	Face B	−1	+1	0	0	Uniform
12	Face B	−1	−1	0	0	Uniform

13	Face B	+1	0	+1	0	Uniform
14	Face B	+1	0	−1	0	Uniform
15	Face B	0	+1	+1	0	Uniform
16	Face B	0	+1	−1	0	Uniform
17	Face B	0	0	+1	+1	Uniform
18	Face B	0	0	+1	−1	Uniform
19	Face B	0	+1	0	+1	Uniform
20	Face B	0	−1	0	+1	Uniform
21	Fill C	−0.5	+0.5	−0.5	+0.5	D-optimal
22	Fill C	+0.5	−0.5	+0.5	−0.5	D-optimal
23	Fill C	+0.5	+0.5	−0.5	−0.5	D-optimal
24	Fill C	−0.5	−0.5	+0.5	+0.5	D-optimal
25	Fill C	0	+0.5	+0.5	−0.5	D-optimal
26	Fill C	+0.5	0	−0.5	+0.5	D-optimal

Table S6. Evaluation_Criteria

Criterion	Meaning	Use in Model Selection
R^2	Goodness of fit on training data	Indicates how much variation the model explains; should not be used

		alone.
Adjusted R^2	R^2 adjusted for the number of predictors	Useful for comparing models with different numbers of terms.
LOOCV R^2	Cross-validated predictive ability	Primary indicator for robustness when sample size is limited.
RMSECV	Root mean squared error from LOOCV	Lower values indicate smaller prediction errors.
VIF	Variance inflation factor	Used to assess multicollinearity among predictors.
Residual diagnostics	Residual plots, Q-Q plots, Cook's distance	Used to check model assumptions and identify influential observations.
